# Cellular origin and characterization of autoantibodies against type I IFNs in COVID-19 and APS-1

**DOI:** 10.1084/jem.20260268

**Published:** 2026-11-03

**Authors:** Rui Sun, Siyu Lin, Luca Simonelli, Benedikt Strunz, Anish Behere, Mattia Pedotti, Tian Xu, Fanglei Zuo, Likun Du, Hui Wan, Sara Lestani, Leire de Campos-Mata, Stelios Vlachiotis, Dorottya Kalmár, Karolinska KI/K COVID-19 Study Group, Anders Sönnerborg, Dorota Religa, Susanna Brighenti, Benedict J. Chambers, Martin Cornillet, Angelica Cuapio, Malin Flodström-Tullberg, Nicole Marquardt, Jagadeeswara Rao Muvva, Anna Norrby-Teglund, Johan K. Sandberg, David Wullimann, Isabel Diaz Lozano, Kristoffer Strålin, Soo Aleman, Hans-Gustaf Ljunggren, Antonio Piralla, Fausto Baldanti, Olle Kämpe, Sophie Bensing, Lennart Hammarström, Nils Landegren, Zexian Zeng, Niklas K. Björkström, Luca Varani, Harold Marcotte, Qiang Pan-Hammarström

**Affiliations:** 1 Division of Immunology, Department of Medical Biochemistry and Biophysics, Karolinska Institutet, Stockholm, Sweden; 2 Peking-Tsinghua Center for Life Sciences, Academy for Advanced Interdisciplinary Studies, Peking University, Beijing, China; 3 Institute for Research in Biomedicine, Università Della Svizzera Italiana, Bellinzona, Switzerland; 4 Department of Medicine Huddinge, Center for Infectious Medicine, Karolinska Institutet, Karolinska University Hospital, Stockholm, Sweden; 5 Science for Life Laboratory, Department of Medical Biochemistry and Microbiology, Uppsala University, Uppsala, Sweden; 6 Department of Infectious Diseases, Karolinska University Hospital, Stockholm, Sweden; 7 Microbiology and Virology Department, Fondazione IRCCS Policlinico San Matteo, Pavia, Italy; 8 Department of Clinical, Surgical, Diagnostic and Paediatric Sciences, University of Pavia, Pavia, Italy; 9 Department of Endocrinology, Karolinska University Hospital, Stockholm, Sweden; 10 Department of Medicine (Solna), Karolinska Institutet, Stockholm, Sweden

## Abstract

Autoantibodies against type I IFNs can severely compromise antiviral defense, yet the mechanisms underlying their development remain elusive. Using high-throughput autoantigen profiling, we observed that a subgroup of severe COVID-19 patients exhibited strong binding to multiple type I IFNs. Integrated single-cell RNA and B cell receptor (BCR) sequencing with functional analysis identified B cell subsets/states enriched for IFN-α2–binding and –neutralizing antibodies in COVID-19. These findings were compared with those from patients with autoimmune polyendocrine syndrome type I, a rare genetic disease characterized by defective immune tolerance and persistent type I IFN autoimmunity. Competition surface plasmon resonance and AlphaFold 3 structural modeling identified three major functional epitope groups and seven predicted structural subclasses among neutralizing antibodies. Our results indicate that IFN-α2 autoreactivity is present in both naive and antigen-experienced B cell compartments, whereas functional IFN-neutralizing antibodies are predominantly associated with memory and atypical B cells, consistent with defective tolerance and affinity maturation-mediated selection of autoreactive B cell clones.

## Teaser

Sun et al. investigate the cellular origins, B cell states, and functional properties of type I IFN–neutralizing autoantibodies associated with severe COVID-19 and APS-1. They show that functional IFN-neutralizing antibodies arise predominantly from subsets of memory and atypical B cells.

## Graphical Abstract

**Figure:**
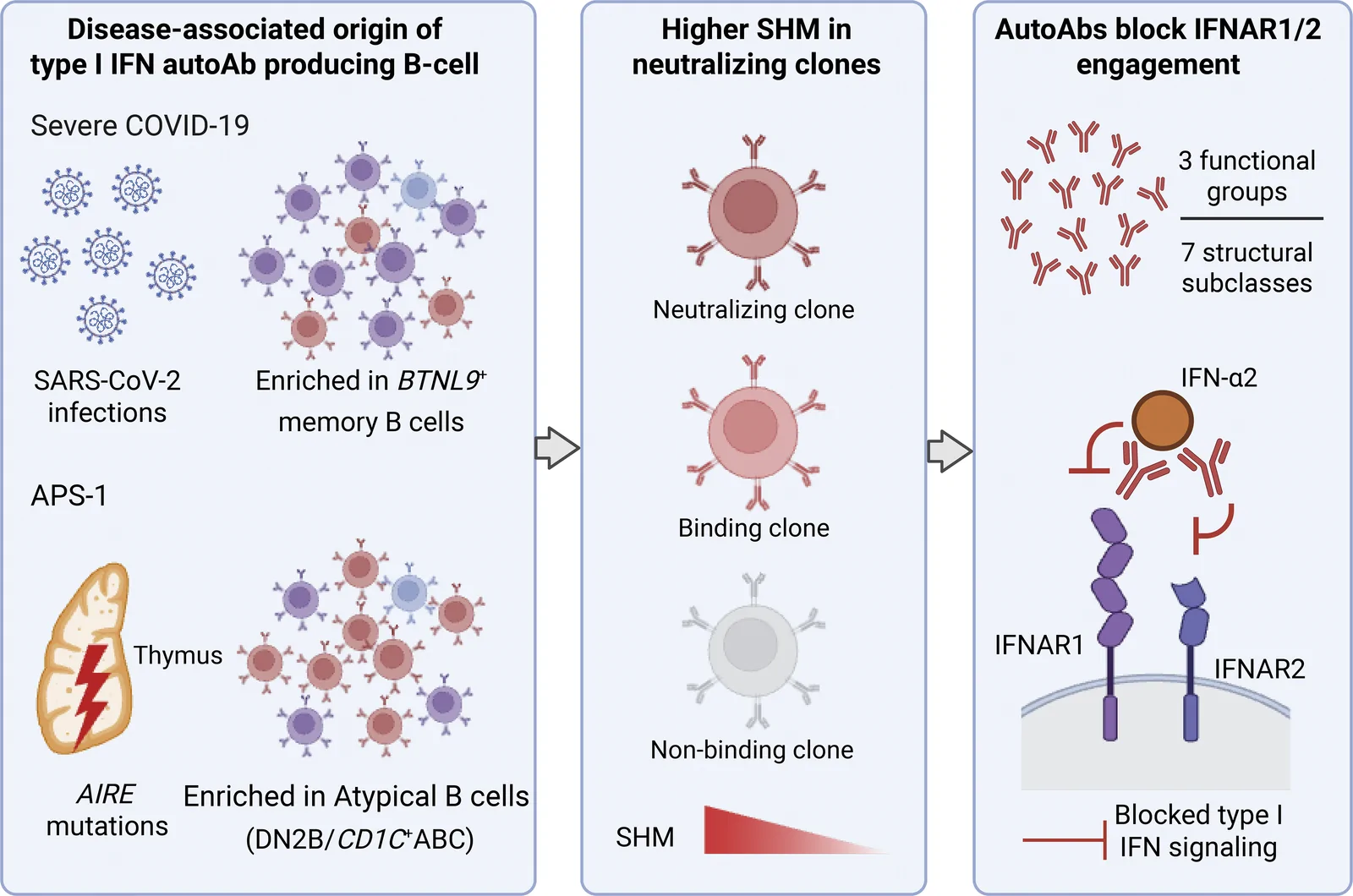

## Introduction

IFNs are cytokines essential for host defense against viral and bacterial infections (McNab et al., 2015). Type I IFNs, produced by most nucleated cells in response to viral infection, coordinate early innate immunity and prime adaptive immune responses. The human type I IFN family includes 13 highly homologous IFN-α subtypes, IFN-β, and additional members such as IFN-ɛ, IFN-τ, IFN-κ, IFN-ω, IFN-δ, and IFN-ζ (Pestka et al., 2004). All type I IFNs signal through the surface IFN-α/β receptor (IFNAR) complex to induce the transcription of IFN-stimulated genes (ISGs) that mediate antiviral and immunomodulatory functions (Baccala et al., 2005; Bennett et al., 2003; Kisand et al., 2008; Pestka et al., 2004).

Anti-cytokine autoantibodies (autoAbs) are detectable at low levels in healthy individuals, where they may contribute to immune regulation and homeostasis (Olsen and Karp, 2014). Not all anti-IFN autoAbs are pathogenic; only those with neutralizing activity can impair IFN signaling and are strongly associated with susceptibility to severe viral infections. High levels of such autoAbs may therefore predispose individuals to increased severity of infections (Sun et al., 2023). During the COVID-19 pandemic, neutralizing autoAbs against type I IFNs were identified in 13.7% of patients with life-threatening disease and in ∼20% of fatal cases (Bastard et al., 2020; Chauvineau-Grenier et al., 2022). The prevalence of these autoAbs increases with age, rising sharply in individuals older than 70 years (Bastard et al., 2021a). Patients harboring such type I IFN autoAbs exhibited reduced ISG expression, impaired antiviral responses, and elevated biomarkers of severe disease (Frasca et al., 2022). These autoAbs are present not only in the circulation but also at mucosal sites, where they can impair early antiviral responses in the upper respiratory tract (Lopez et al., 2021). Moreover, neutralizing autoAbs against type I IFNs have been linked to severe breakthrough infections (Bastard et al., 2023).

Pathogenic type I IFN autoAbs have been identified across a broad range of single-stranded RNA viral infections. High prevalence has been reported in arbovirus-associated diseases, including ∼40% of West Nile virus encephalitis cases (Gervais et al., 2023), 35% of life-threatening adverse events following yellow fever vaccination (Bastard et al., 2021b), and 10% of severe tick-borne encephalitis cases (Gervais et al., 2024b), as well as in severe infections caused by Powassan, Usutu, and Ross River viruses (Gervais et al., 2024a). Additionally, neutralizing autoAbs against type I IFNs have been implicated in ∼5% of critical influenza pneumonia cases (Zhang et al., 2022), 20% of Middle East respiratory syndrome cases (Alotaibi et al., 2023), a recent fatal case of H5N1 avian influenza (Zhang et al., 2026), three cases of encephalitis following vaccination with a live-attenuated chikungunya vaccine (Gervais et al., 2026a), and 37.5% of fulminant viral hepatitis cases triggered by the double-stranded DNA virus herpes simplex virus (Gervais et al., 2026b).

Genetic determinants of type I IFN autoimmunity have been identified in autoimmune polyendocrine syndrome type I (APS-1), an inborn error of immunity (IEI) caused by biallelic *AIRE* mutations that impair development of central tolerance in the thymus (Constantine and Lionakis, 2019; Husebye et al., 2018). APS-1 patients harbor high-titer neutralizing autoAbs against type I IFNs and exhibit a markedly increased risk of severe viral infections (Bastard et al., 2021c; Hetemäki et al., 2021). Although a small number of type I IFN–autoAb–positive COVID-19 cases can be explained by inborn errors of thymic T cell tolerance, including defects in the alternative NF-κB pathway or loss-of-function variants in the gene encoding NF-κB essential modulator (Le Voyer et al., 2023; Rosain et al., 2024), the mechanisms driving the development of type I IFN autoAbs in most patients remain elusive. In particular, the cellular origins and molecular characteristics of these pathogenic autoAbs remain poorly understood. A recent study demonstrated that B cells producing type I IFN–neutralizing antibodies from patients with severe COVID-19 are largely indistinguishable from those found in individuals with inherited defects in T cell tolerance and generate high-affinity neutralizing antibodies through extensive somatic hypermutation (SHM) (Fournier et al., 2026). Another recent study showed that the abundance of autoAbs in COVID-19 was generally associated with the expansion of IgD^−^CD27^−^ double-negative atypical memory B cells (Bm; DN2B) (Yuan et al., 2026).

Here, we integrated single-cell transcriptomics and BCR analysis with functional and structural characterization of recombinant antibodies to define the cellular basis, differentiation states, and molecular features of type I IFN–neutralizing autoAbs in severe COVID-19 and APS-1 patients. We showed that neutralizing antibody clones followed distinct B cell maturation pathways in these patients, with COVID-19–associated clones predominantly derived from Bm, whereas APS-1–associated clones originated primarily from atypical B cells (ABCs). We further classified the neutralizing antibodies into three major functional epitope groups and seven predicted structural epitope subclasses using competition surface plasmon resonance (SPR) assays and AlphaFold-based modeling.

## Results

### High-throughput autoantigen profiling in severe COVID-19 and APS-1 patients

Nine severe COVID-19 patients who had previously been reported to harbor autoAbs against IFN-α (anti-IFN autoAb positive [aIFN^+^]) (Strunz et al., 2024) were included in this study (Tables S1 and S2). The median age was 61.5 years (range, 50–91 years). Blood samples were collected between April and December 2020, at a median of 15 days after symptom onset (range, 9–28 days). To characterize the autoAb repertoires and confirm the presence of type I IFN autoAbs, serum samples from eight patients were reanalyzed using a customized protein microarray comprising ∼500 antigens (Behere et al., 2025), including type I, II, and III IFNs, cytokines, chemokines, intracellular autoantigens, and viral antigens. Serum or plasma samples from 14 age- and sex-matched severe COVID-19 patients lacking anti-type I IFN autoAbs (anti-IFN autoAb negative [aIFN^−^]), 4 mild COVID-19 patients who were aIFN^−^, 5 APS-1 patients, 5 noninfected, non-vaccinated healthy controls (NC) recruited between May 2020 and May 2021, and 12 additional healthy donors recruited between September 2022 and September 2025 served as controls (Tables S1 and S2).

**Figure 1.**
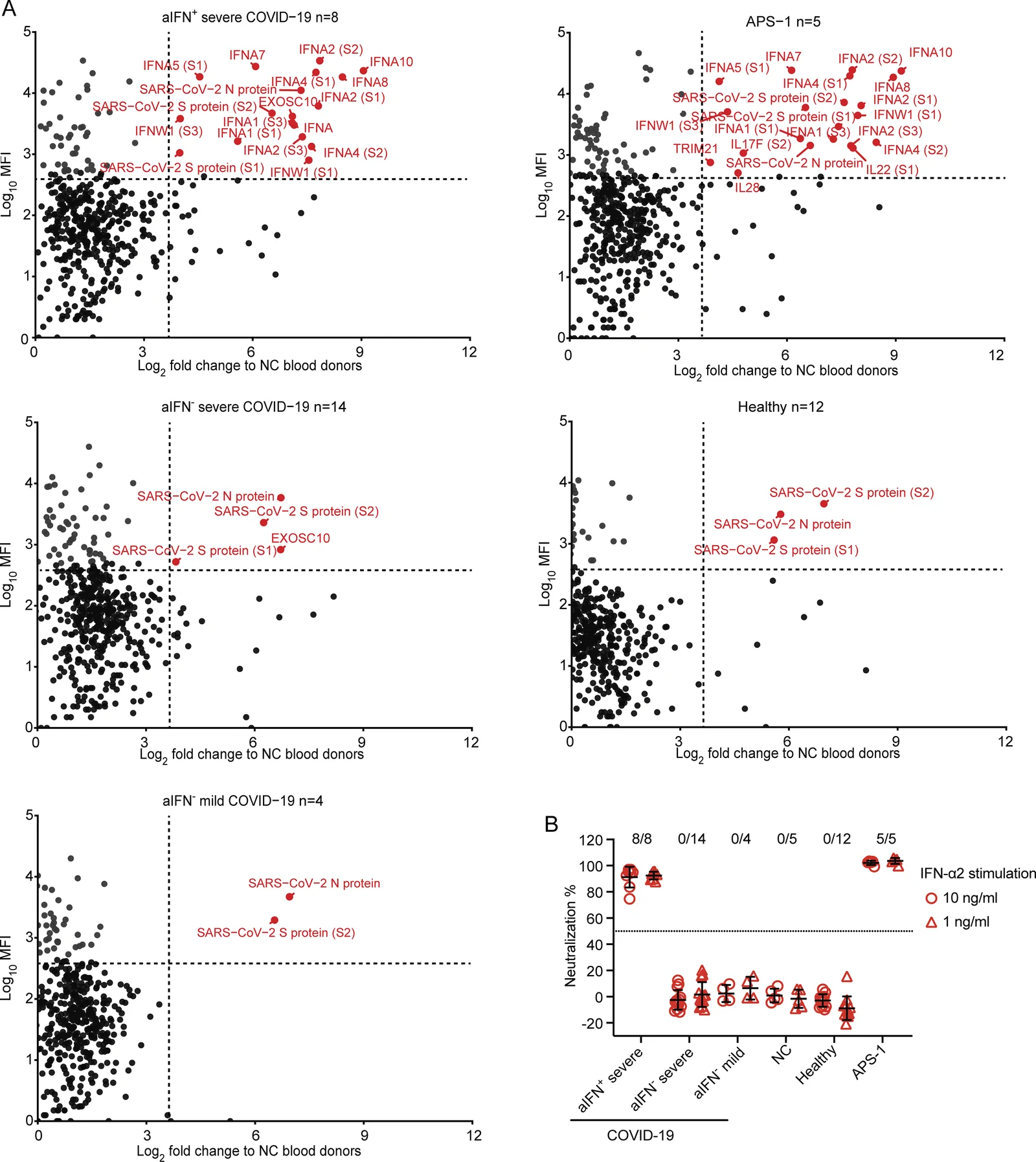
**Cytokine and self-antigen autoreactivity in COVID-19 and APS-1 patients. (A)** AutoAb reactivity against cytokines, chemokines, classical autoimmune antigens, and viral antigens was assessed using a customized protein array in patients with aIFN^+^ severe COVID-19 (*n* = 8), aIFN^−^ severe COVID-19 (*n* = 14), aIFN^−^ mild COVID-19 (*n* = 4), patients with APS-1 (*n* = 5), and healthy blood donors (*n* = 12). Data are shown as log_2_ fold change relative to the mean signal from noninfected, non-vaccinated controls (NC, *n* = 5). Positivity was defined as the log_2_ fold change ≥3.5 relative to the mean of the NC group and MFI ≥500 arbitrary units (log_10_ MFI ≥2.699). Dashed lines indicate the positivity thresholds. Data are representative of two independent experiments. **(B)** Neutralization of IFN-α2–induced signaling was assessed using HEK-Blue IFN-α/β reporter cells. Serum or plasma samples from patients and controls were incubated with IFN-α2 at 10 or 1 ng ml^−1^ before being added to the reporter cells. SEAP reporter activity was then measured. Data are presented as mean ± SD. The dashed line indicates the 50% neutralization threshold. Numbers above each group indicate the number of samples exceeding this threshold at 10 and 1 ng ml^−1^ IFN-α2, respectively. Data are representative of two independent experiments.

**Figure S1.**
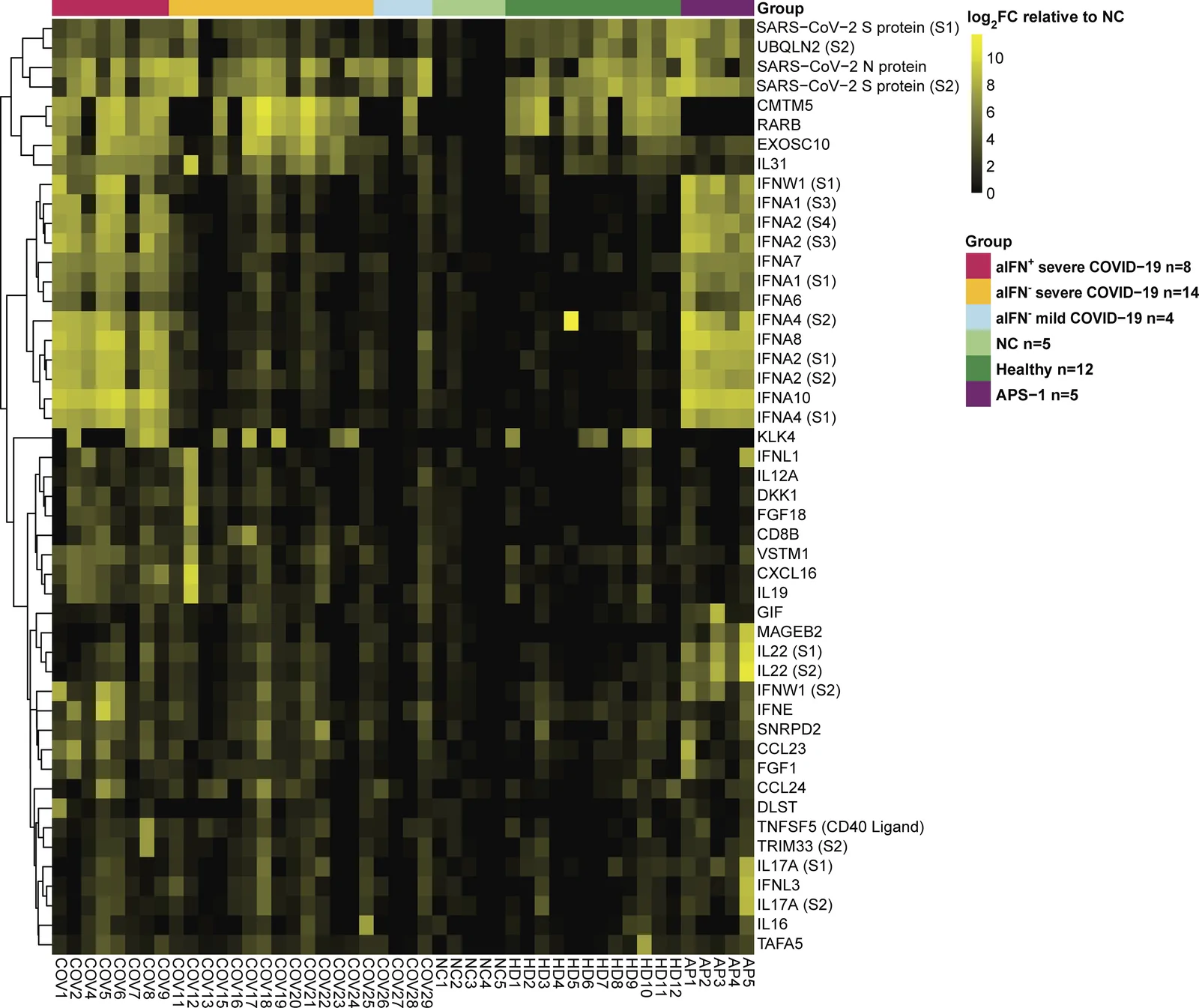
**Related to Fig. 1. Cytokine and self-antigen autoreactivity at the individual level.** Cytokine and self-antigen autoreactivity profiling of patients with aIFN^+^ severe COVID-19 (*n* = 8), aIFN^−^ severe COVID-19 (*n* = 14), aIFN^−^ mild COVID-19 (*n* = 4), healthy blood donors (*n* = 12), and APS-1 (*n* = 5) using a customized protein array. Data are presented as log_2_ fold change relative to the mean signal of NCs (no SARS-CoV-2 infections, no COVID-19 vaccinations; *n* = 5). Positivity was defined as a log_2_ fold change ≥6.644 relative to the mean of the NC group and MFI ≥500 arbitrary units (log_10_ MFI ≥2.699). Antigens meeting both thresholds are shown. The more stringent log_2_ fold change threshold was applied for visualization of individual-level autoreactivity profiles.

As expected, sera from patients with aIFN^+^ COVID-19 and APS-1 showed strong binding to multiple type I IFNs, including several IFN-α subtypes and IFN-ω, whereas little or no reactivity was detected in aIFN^−^ COVID-19 patients or in healthy donors (Fig. 1 A and Table S3). At the cohort level, no significant increase in binding to IFN-β, IFN-ε, IFN-κ, or type II or type III IFNs was observed in either the COVID-19 or APS-1 groups, although occasional reactivity to IFN-ε, IFN-λ1, and IFN-λ3 was detected in individual patients with APS-1 and severe COVID-19 (Fig. S1 and Table S3). APS-1 patients furthermore showed specific autoreactivity to IL-17 and IL-22. Beyond type I IFNs, aIFN^+^ severe COVID-19 patients exhibited additional autoreactivity targeting classical self-antigens like EXOSC10 (Fig. 1 A and Table S3) and autoreactivity to multiple cytokines and chemokines, including IL-31, IL-19, and CXCL16 in some individuals (Fig. S1 and Table S3). Although some of these autoreactivities were also detected in aIFN^−^ severe COVID-19 patients, very few autoreactivities were observed in patients with mild disease and in healthy donors. These observations are consistent with previous reports showing that dysregulated B cell responses and the generation of autoAbs occur in severe COVID-19 cases (Chang et al., 2021; Wang et al., 2021; Woodruff et al., 2022). Notably, antibody reactivity against the SARS-CoV-2 nucleocapsid (N) protein and spike protein was not detected in the NC group but was detected in all patient groups and some healthy donors collected during the later stage of the pandemic, consistent with their history of infections and vaccinations (Fig. 1 A and Fig. S1).

To assess the functional impact of type I IFN autoAbs, we performed a neutralization assay using a HEK-Blue IFN-α/β reporter cell line (Behere et al., 2025). Sera from patients with aIFN^+^ severe COVID-19 and APS-1 strongly inhibited IFN-α2-induced signaling at both 10 and 1 ng ml^−1^. In contrast, samples from patients with aIFN^−^ severe or mild COVID-19 and from healthy donors showed no detectable neutralization, consistent with previous studies (Bastard et al., 2021c; Levin, 2006; Meager et al., 2006) (Fig. 1 B).

Altogether, these findings indicate that aIFN^+^ severe COVID-19 patients exhibit potent neutralization of IFN-α2, along with reactivity against additional self-antigens. A similarly broad autoreactive profile was observed in APS-1 patients, although this condition is characterized by additional, distinct autoAb specificities targeting T helper 17 cell-related cytokines.

### Single-cell profiling of B cells and other immune cell compartments in severe COVID-19 and APS-1 patients

**Figure 2.**
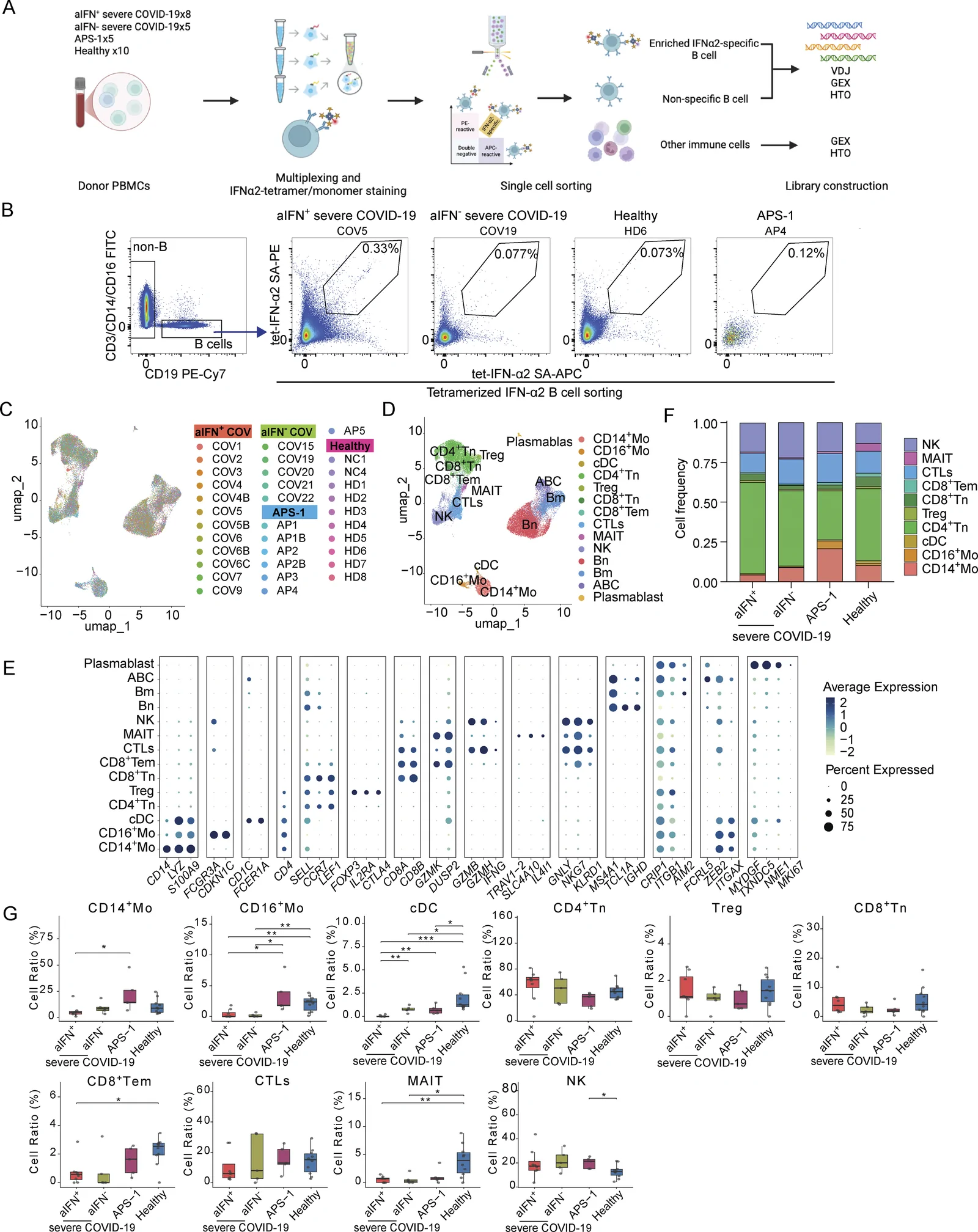
**Altered immune cell composition in aIFN** ^**+**^ **severe COVID-19 patients compared with control cohorts. (A)** Schematic overview of the experimental design. Four cohorts were included: aIFN^+^ severe COVID-19, aIFN^−^ severe COVID-19, APS-1, and healthy controls. **(B)** Enrichment and flow-cytometric identification of tetramer-positive B cells using tetramerized IFN-α2 antigen from representative donors. Representative flow cytometry plots from five independent experiments are shown. **(C and D)** UMAP plots of all single cells colored by donor (C) and by annotated immune cell populations (D). **(E)** Dot plot illustrating the expression of canonical marker genes used to annotate immune cell populations. Dot size represents the percentage of cells expressing each gene, and color intensity indicates the mean expression level. **(F and G)** Proportional representation of major non–B cell immune populations across cohorts. Data are shown as relative cell type frequencies at the cohort level (F) and individual donor distributions (G). In G, the central line indicates the median, and the boxes represent the interquartile range. Statistical significance was determined using the Wilcoxon rank-sum test; *P < 0.05, **P < 0.01, and ***P < 0.001.

**Figure S2.**
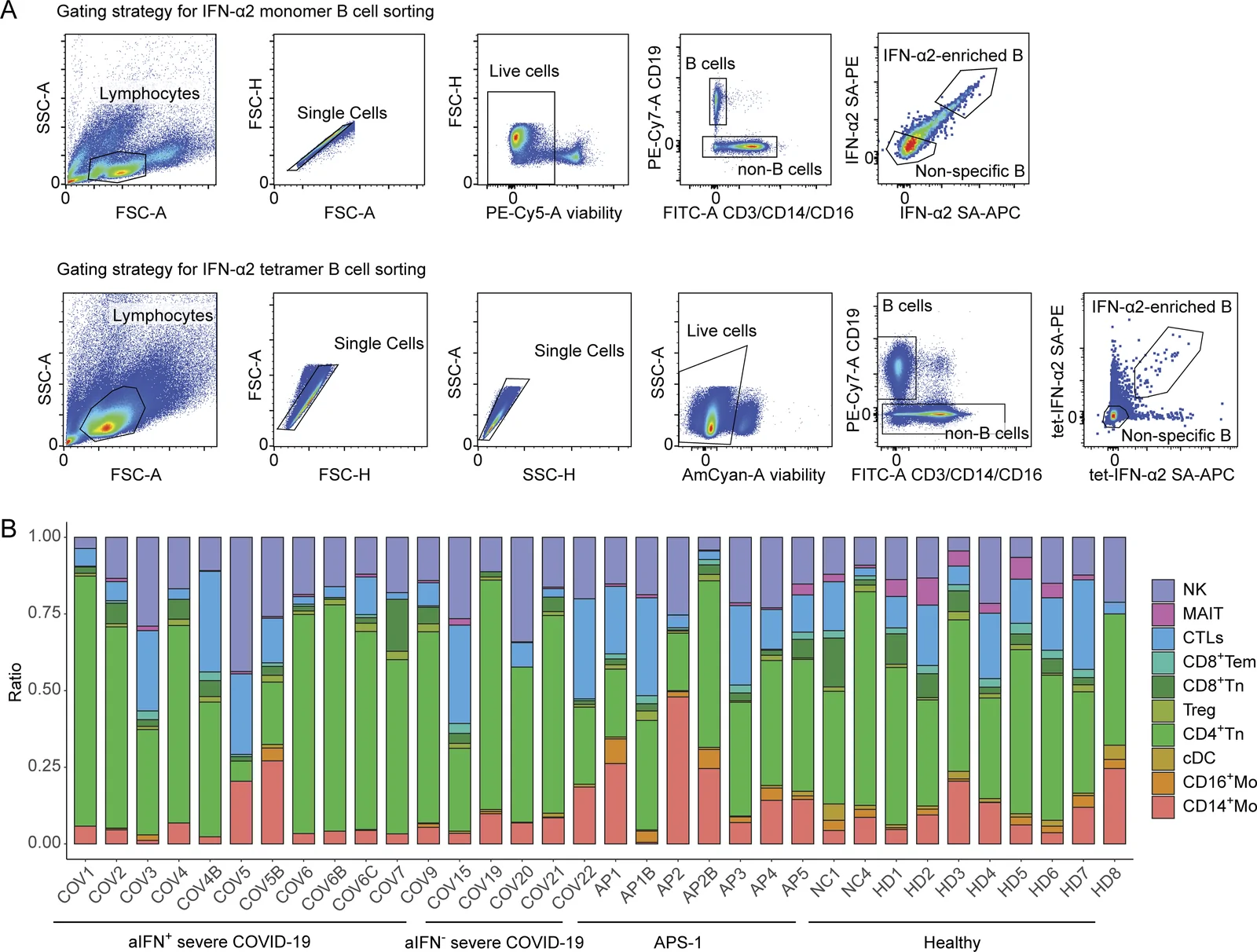
**Related to Fig. 2. (A)** Gating strategy for IFN-α2 monomer and tetramer-based B cell sorting. Flow cytometric gating used to isolate non–B immune cells, IFN-α2–monomer– and tetramer–positive B cells, as well as IFN-α2–monomer– and tetramer–negative B cells for downstream scRNA-seq and scBCR-seq analyses. **(B)** Proportions of major non–B immune cell subsets for each donor. Donors were stratified by cohort (aIFN^+^ severe COVID-19, aIFN^−^ severe COVID-19, APS-1, and healthy donors).

To assess alterations in the overall immune landscape and to characterize IFN-α2–specific B cells in aIFN^+^ COVID-19 patients, we performed single-cell RNA sequencing (scRNA-seq) of B cells identified by IFN-α2 monomer- or tetramer-staining (hereafter referred to as monomer- or tetramer-positive B cells), along with the remaining B cells and non-B immune cells isolated from peripheral blood mononuclear cells (PBMCs) (Fig. 2 A and Fig. S2). Although tetramer-positive cells were identified in all groups, higher frequencies of IFN-α2 tetramer-positive B cells were observed in patients with aIFN^+^ severe COVID-19 and APS-1 than in aIFN^−^ severe COVID-19 patients and healthy donors (Fig. 2 B), supporting the utility of the IFN-α2 tetramer-staining for enriching B cell populations containing IFN-binding clones. In parallel, we performed single-cell BCR sequencing (scBCR-seq) on the B cell compartments.

PBMC samples were obtained from eight patients with aIFN^+^ severe COVID-19, with the first sample collected during the acute phase (seven samples collected between 9 and 28 days post-symptom onset; one sample for which the exact timing was unavailable) (Strunz et al., 2024). Longitudinal PBMC samples were available from three patients; two were sampled at second and third time points within 30 days of symptom onset, whereas one was sampled at a second time point on day 214 after symptom onset. In addition, PBMCs from 5 age-matched aIFN^−^ severe COVID-19 patients, 5 APS-1 patients, and 10 healthy donors (including two NCs) were included for comparison (Fig. 2 A, Tables S1 and S2). For each scRNA-seq library, cells from five to eight donors were pooled. To preserve donor identity, PBMCs were multiplexed using TotalSeq hashtag antibodies in combination with IFN-α2 and surface-marker staining.

After quality control and filtering, we obtained 38,242 high-quality single-cell transcriptomes from 34 samples derived from 28 individuals, comprising 17,806 B cells, including 1,541 IFN-α2-tetramer–positive B cells, and 20,436 non-B immune cells. Data from different sequencing batches and donors were subjected to principal component analysis (PCA), followed by batch correction. The integrated dataset was then projected using Uniform Manifold Approximation and Projection (UMAP) for dimensionality reduction and visualization (Fig. 2 C). Unsupervised clustering identified 14 clusters, including 4 major B cell clusters: naive B cell (Bn), Bm, ABC, and plasmablast, as well as 10 non-B immune cell clusters: natural killer cell, mucosal-associated invariant T cell (MAIT), cytotoxic T lymphocyte, CD8^+^ effector memory T cell (CD8^+^Tem), CD8^+^ naive T cell, regulatory T cell, CD4^+^ naive T cell, conventional dendritic cell (cDC), CD16^+^ monocyte (CD16^+^Mo), and CD14^+^ monocyte (CD14^+^Mo) (Fig. 2, D and E). Cluster annotations were supported by expression of canonical marker genes of major immune cell lineages and subsets.

We next compared the frequencies of non-B immune cell subsets across patient and control cohorts (Fig. 2, F and G; and Fig. S2 B). Severe COVID-19 cohorts showed altered cellular composition compared with healthy donors, including reduced frequencies of CD16^+^Mo, cDCs, CD8^+^Tem, and MAIT cells (Fig. 2 G), largely consistent with previous reports (Parrot et al., 2020; Sanchez-Cerrillo et al., 2020; Schulte-Schrepping et al., 2020). APS-1 patients showed similar trends, although no reduction in CD16^+^Mo was observed. Notably, the proportions of cDCs were reduced in all disease groups compared with healthy donors, with the lowest frequencies observed in patients with aIFN^+^ severe COVID-19.

### IFN-α2-tetramer–positive B cells are enriched in selected B cell subsets

**Figure 3.**
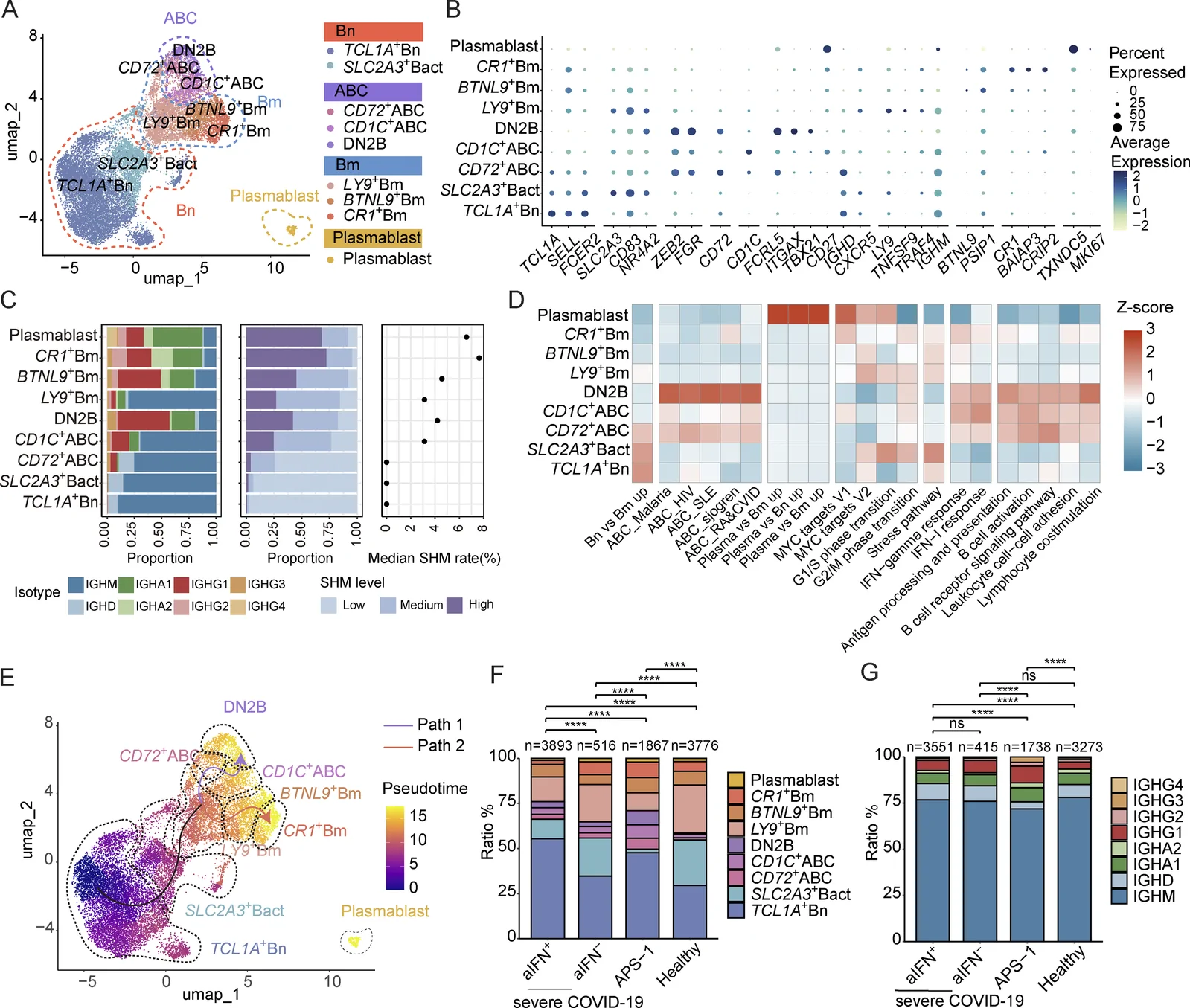
**B cell subcluster distribution and transcriptional features in patients with aIFN** ^**+**^ **severe COVID-19, aIFN^−^** **severe COVID-19, and APS-1. (A)** UMAP visualization of B cell subclusters. **(B)** Dot plot showing the expression of canonical marker genes used for B cell subcluster annotation. Dot size represents the percentage of cells expressing each gene, and color intensity indicates the mean expression level. **(C)** B cell isotype proportions and SHM frequencies across B cell subclusters. **(D)** B cell–related signature scores across B cell subclusters. Z-scores indicate the relative strength of pathway activation compared with background expression. **(E)** Trajectory analysis illustrating inferred transcriptional relationships among B cell states. Path 1 depicts the inferred ABC trajectory, whereas path 2 represents the inferred Bm trajectory. Pseudotime scores indicate each cell’s relative position along the inferred trajectory. **(F and G)** Proportions of B cell subclusters (F) and IGH isotype distributions (G) among sorted B cells across the four cohorts. Statistical significance was determined by comparing overall B cell subset distributions (F) or IGH isotype distributions (G) between cohorts using two-sided Fisher’s exact tests with Benjamini–Hochberg correction for multiple comparisons; ns = not significant (P > 0.05), ****P < 0.0001.

To increase the resolution of the B cell compartment, we subsequently reclustered all B cells and identified nine transcriptionally distinct subclusters/states within the four major B cell clusters, including Bn, Bm, ABC, and plasmablast (Fig. 3, A–C). Within the naive B cell cluster, the *SLC2A3*^*+*^ activated B cell (Bact) subcluster showed enrichment of cell cycle and stress pathways, whereas the *TCL1A*^*+*^ Bn subcluster displayed a relatively quiescent profile (Fig. 3 D). Within the memory B cell cluster, the *LY9*^+^ Bm subcluster shared transcriptional features with the *SLC2A3*^+^ Bact subcluster, whereas the *CR1*^*+*^ Bm and *BTNL9*^*+*^ Bm subclusters exhibited MYC and IFN-γ response signatures (Fig. 3 D). Three ABC subsets displayed transcriptional programs associated with autoimmune and infection-associated settings (Yang et al., 2024), marked by enrichment of IFN-γ and type I IFN responses, B cell activation, BCR signaling, and antigen-processing pathways.

To delineate transcriptional state relationships among B cell subsets, we reconstructed pseudotime trajectories and identified two major paths corresponding to Bm and ABC states (Fig. 3 E). These trajectories represent inferred transcriptional relationships and do not establish developmental lineage relationships. *TCL1A*^*+*^ Bn population was used as the inferred root for both trajectories (Fig. 3 E), with cells distributed along paths consistent with an inferred progression through *SLC2A3*^*+*^ Bact intermediates. Thereafter, B cells were distributed along two inferred trajectories, corresponding to Bm and ABC. The Bm trajectory progressed through *LY9*^*+*^ Bm, *BTNL9*^*+*^ Bm, and *CR1*^*+*^ Bm states, whereas the ABC trajectory encompassed *CD72*^*+*^ ABC, *CD1C*^*+*^ ABC, and DN2B states. Both pathways were accompanied by increasing frequencies of class switch recombination and SHM, consistent with progressive B cell maturation (Fig. 3 C).

Focusing on the tetramer sorting-generated dataset, patients with aIFN^+^ severe COVID-19 showed an increased representation of Bn subset, with the highest proportion of *TCL1A*^+^ Bns observed in this cohort (55.5%). In contrast, expansion of ABCs was observed in all three disease cohorts, with the greatest enrichment of the three ABC subsets (DN2B, 7.8%; *CD1C*^*+*^ ABC, 7.5%; and *CD72*^*+*^ ABC, 6.0%) and the highest frequency of class-switched Ig isotypes observed in patients with APS-1 (24.4%) (Fig. 3, F and G; and Table S4).

**Figure S3.**
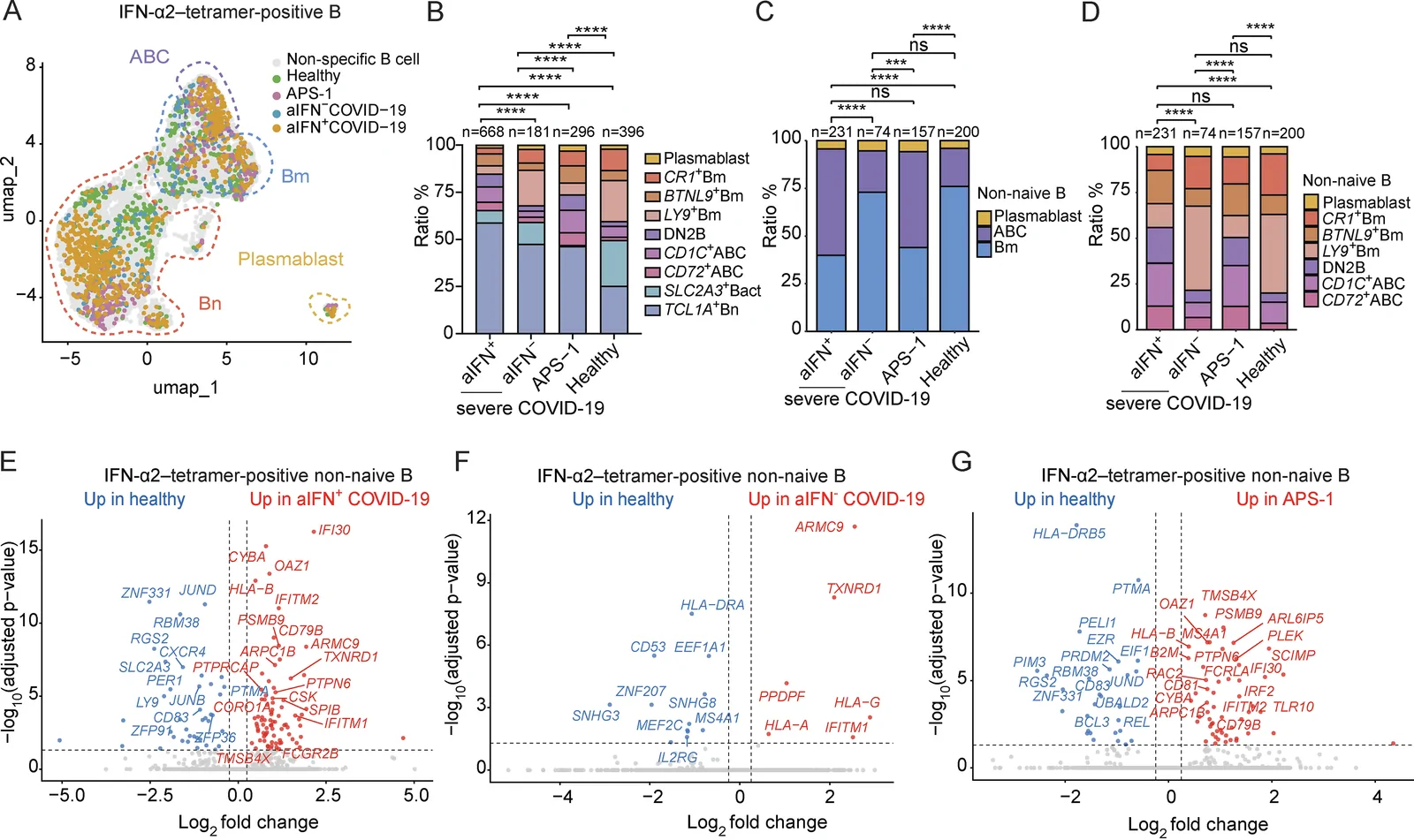
**Related to Fig. 3. (A)** UMAP visualization of cohort-associated IFN-α2-tetramer–positive B cell distributions across aIFN^+^ severe COVID-19, aIFN^−^ severe COVID-19, APS-1, and healthy donors. **(B)** Comparison of the proportions of the nine B cell subclusters among IFN-α2-tetramer–positive B cells across aIFN^+^ severe COVID-19, aIFN^−^ severe COVID-19, APS-1, and healthy cohorts. **(C and D)** Comparison of the proportions of Bms, ABCs, and plasmablasts (C) and seven subclusters (D) among IFN-α2-tetramer–positive, non-naive B cells across aIFN^+^ severe COVID-19, aIFN^−^ severe COVID-19, APS-1, and healthy cohorts. **(E–G)** DEGs in IFN-α2-tetramer–positive non-naive B cells in aIFN^+^ COVID-19 (E), aIFN^−^ COVID-19 (F), and APS-1 (G), compared with healthy donors. DEGs were defined as p_adj <0.05 and |log2 (fold change)| >0.25. Statistical significance was determined by comparing overall B cell subset distributions (B–D) between cohorts using two-sided Fisher’s exact tests with Benjamini–Hochberg correction for multiple comparisons; ns = not significant (P > 0.05), ***P < 0.001, and ****P < 0.0001.

We next mapped the tetramer-positive cells across different B cell subclusters (Fig. S3 A). Overall, tetramer-positive B cells exhibited a distribution similar to that of the global B cell compartment, with further enrichment of *TCL1A*^+^ Bns (58.8 vs. 47.5%) and ABCs (19.3 vs. 8.9%) among patients with aIFN^+^ severe COVID-19 compared with aIFN^−^ severe COVID-19 (Fig. S3 B). Focusing on the antigen-experienced, non-naive B cell compartment, aIFN^+^ severe COVID-19 and APS-1 patients showed a similar preference for *BTNL9*^+^ Bms (18.2 and 17.2%, respectively) and ABC subclusters (55.8 and 50.3%, respectively), whereas aIFN^−^ severe COVID-19 patients displayed a subset composition closer to that observed in healthy donors (Fig. S3, C and D; and Table S4).

IFN-α2-tetramer–positive, non-naive B cells from both aIFN^+^ disease groups also shared upregulated genes and pathways related to humoral immune responses and BCR signaling (*CD79B, PTPN6*), antigen presentation (*HLA-B, PSMB9,* and *IFI30*), NADPH oxidase (*CYBA*), and type I and type II IFN responses, including *IFITM2*, *IFITM1*, and *IRF2*, compared with healthy donors (Fig. S3, E–G). In contrast, few differentially expressed genes (DEGs) were detected in IFN-α2-tetramer–positive cells from aIFN^−^ COVID-19 patients.

Together, high-resolution analysis identified nine transcriptionally distinct B cell subclusters/states that differed in abundance across disease and control groups. The IFN-α2 tetramer–positive B cell population, which is enriched for but not exclusively composed of IFN-α2–reactive B cells, was preferentially distributed within selected Bm and ABC subclusters in patients with aIFN^+^ severe COVID-19 and APS-1.

### B cells encoding IFN-α2–binding antibodies showed diverse cellular origins and immunoglobulin heavy-chain variable region characteristics

**Figure 4.**
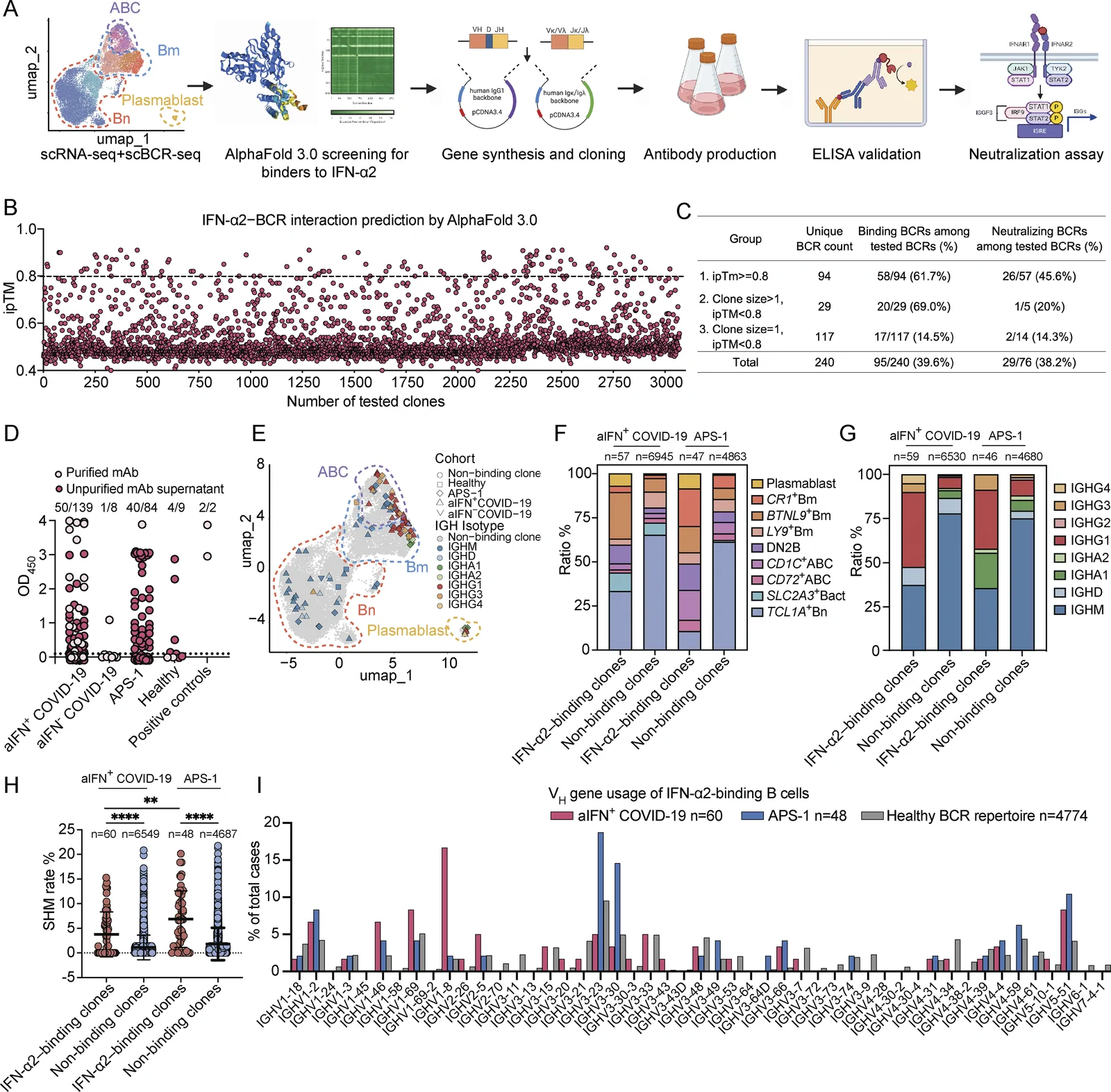
**Cloning and characterization of IFN-α2–binding clones. (A)** Workflow for IFN-α2–binding B cell screening, antibody production, and functional characterization. **(B)** ipTM scores for AlphaFold 3-predicted interactions between IFN-α2 and BCRs from sorted B cells. **(C)** BCR groups selected for recombinant antibody production. **(D)** ELISA validation of IFN-α2 binding by purified mAbs at 1 μg ml^−1^ and 293FT cell culture supernatants expressing recombinant antibodies; OD_450_ = 0.1 was used as the binding cutoff. Data are pooled from four independent experiments. Tetramer-derived clones were validated in two to three independent ELISA experiments, whereas monomer-derived clones were initially identified in a single supernatant screen and subsequently reexpressed, purified, and retested by ELISA. **(E)** UMAP visualization of confirmed IFN-α2–binding clones (defined as B cell clones encoding recombinant antibodies with detectable IFN-α2 binding by ELISA) colored by cohort and IGH isotype. **(F and G)** Comparison of B cell subclusters (F) and immunoglobulin isotypes (G) between IFN-α2–binding and non-binding clones from individuals with aIFN^+^ COVID-19 and APS-1. **(H)** Comparison of SHM frequencies between IFN-α2–binding clones and non-binding clones from individuals with aIFN^+^ COVID-19 and APS-1. Data are presented as mean ± SD. **(I)** IGHV gene usage among IFN-α2–binding clones from individuals with COVID-19 or APS-1, compared with BCR repertoire from healthy donors. Differences in clone numbers between panels reflect analysis-specific inclusion criteria, including clonotype collapsing, paired BCR sequence availability, and experimental validation. Statistical significance was determined using the Wilcoxon rank-sum test; ns = not significant (P > 0.05), **P < 0.01, and ****P < 0.0001.

To further identify and functionally characterize potential IFN-α2–binding and –neutralizing clones, we selected and cloned a panel of unique BCRs (*n* = 240) from various B cell populations (Fig. 4, A–C). These primarily included IFN-α2-tetramer–positive B cells, as well as cells showing high or intermediate signal during monomer-based enrichment. BCR sequence information was extracted from the scBCR-seq dataset. The resulting recombinant mAbs were expressed in an IgG1 format, irrespective of their original heavy-chain isotype to enable standardized comparison across clones. They were then assessed for IFN-α2 binding by ELISA and for biological activity using type I IFN neutralization assays (Fig. 4 A). Two commercial anti-IFN-α2 mAbs, sifalimumab and rontalizumab, served as positive controls.

The cloned 240 BCRs were stratified into three groups. Group 1 comprised 94 candidate IFN-α2–binding BCRs predicted by AlphaFold 3, defined by interface-predicted Template Modeling (ipTM) values ≥0.8, a high-confidence threshold for predicted antibody–antigen interface accuracy (Fig. 4 B) (Abramson et al., 2024). Altogether, we screened 3,021 BCR sequences, and the majority of BCRs with an ipTM value ≥0.8 were derived from IFN-α2-tetramer–positive B cell populations (65 of 94, 69.1%), whereas an additional 28 clones were identified from IFN-α2-monomer–enriched cells and one from non-enriched B cells. Given the known limitations of AlphaFold 3 in predicting antigen–antibody interactions (Abramson et al., 2024; Yin and Pierce, 2024), we included two additional groups of BCRs for antibody gene synthesis and expression. Group 2 included 29 BCRs derived from B cell clones with ipTM values <0.8 and clone size >1, defined by the presence of at least two identical clonal groups, while group 3 comprised 117 BCRs with ipTM values <0.8 and clone size = 1. Altogether, 293FT antibody production culture supernatants or purified mAbs from 58 of 94 clones (61.7%) in group 1, 20 of 29 clones (69.0%) in group 2, and 17 of 117 clones (14.5%) in group 3 tested positive for binding to IFN-α2 (Fig. 4 C).

For all downstream analyses, IFN-α2–binding clones were defined functionally as BCRs whose recombinant antibodies showed detectable IFN-α2 binding by ELISA, irrespective of neutralizing activity. Non-binding clones comprised ELISA-negative recombinant antibodies, as well as BCRs derived from IFN-α2-tetramer– and monomer–negative B cells that were not subjected to ELISA testing. Among the 240 antibodies tested, 50 of 139 (36.0%) from aIFN^+^ severe COVID-19 patients, 1 of 8 (12.5%) from aIFN^−^ severe COVID-19 patients, 40 of 84 (47.6%) from APS-1 patients, and 4 of 9 (44.4%) from healthy donors bound type I IFNs, as assessed by ELISA (Fig. 4 D). Next, the IFN-α2–binding clones, color-coded by cohort and IGH isotypes, were projected and visualized using UMAP. B cells carrying identical IFN-α2–binding BCRs were included in this analysis (Fig. 4 E). IFN-α2–binding clones were detected across all major B cell clusters, including Bn, Bm, ABC, and plasmablast populations (Fig. 4 E).

Cohort-associated patterns emerged: aIFN^+^ severe COVID-19–derived IFN-α2–binding clones spanned all major B cell clusters, with 43.9% mapping to naive B cell states (Fig. 4 F). In contrast, APS-1–derived IFN-α2–binding clones were predominantly detected in mature B cell compartments, particularly Bm (42.6%) and ABC (38.3%), whereas only a minority mapped to Bn (10.6%) and plasmablast (8.5%) populations (Fig. 4 F). In aIFN^−^ COVID-19 patients and healthy donors, IFN-α2–binding clones were rare and limited to the Bn and Bm subsets; therefore, they were excluded from subsequent statistical comparisons (Fig. 4, D–E).

Compared with non-binding B cell clones, IFN-α2–binding clones from both aIFN^+^ disease groups showed enrichment of antigen-experienced, non-naive subsets, most notably *BTNL9*^*+*^ Bm in aIFN^+^ COVID-19 (26.3 vs. 7.7%) and DN2B and *CD1C*^*+*^ ABC populations in APS-1 (14.9 vs. 6.0% and 17.0 vs. 6.5%, respectively) (Fig. 4 F; Table S4).

Binding clones also showed more frequent usage of the IGHG1 isotype compared with non-binding clones, with frequencies of 42.4 versus 6.4% in aIFN^+^ COVID-19 and 32.6 versus 9.0% in APS-1, together with higher levels of SHM (Fig. 4, G and H; and Table S4). In APS-1 patients, IFN-α2–binding clones additionally displayed increased usage of the IGHA1 isotype (19.6 vs. 6.1%; Fig. 4 G, Table S4). Nevertheless, IFN-α2–binding BCRs were also detected in Bns expressing IGHM or IGHD across all cohorts (Fig. 4, E–G). These findings suggest that individuals harbor both memory and naive or germline BCR repertoires capable of recognizing IFN-α2.

Next, we examined immunoglobulin heavy-chain variable region (*IGHV*) gene usage among IFN-α2–binding clones. The repertoire exhibited diverse *IGHV* gene utilization across the *IGHV1*–*IGHV5* gene families (Fig. 4 I). Several *IGHV* genes exhibited disease-associated enrichment. *IGHV1–8, IGHV1–46*, and *IGHV1–69* were overrepresented in aIFN^+^ severe COVID-19 patients, whereas *IGHV3–23* and *IGHV3–30* were preferentially enriched in APS-1 patients (Fig. 4 I). In addition, increased usage of *IGHV5–51* was observed in both patient groups.

In summary, IFN-α2*–*binding clones arose from diverse B cell lineages and *IGHV* repertoires, suggesting that IFN reactivity reflects broad precursor pools rather than a single expanded clone. These validated IFN-α2*–*binding clones exhibited disease-specific cellular distributions: those from COVID-19 patients were derived from both naive-like and memory B cell states, whereas those from APS-1 patients were enriched in Bm and ABC states, consistent with potentially prolonged exposure to chronic inflammatory signaling in APS-1 patients.

### IFN-α2-neutralizing clones showed convergent cellular origin, biased IGHV gene usage, and increased SHM

**Figure 5.**
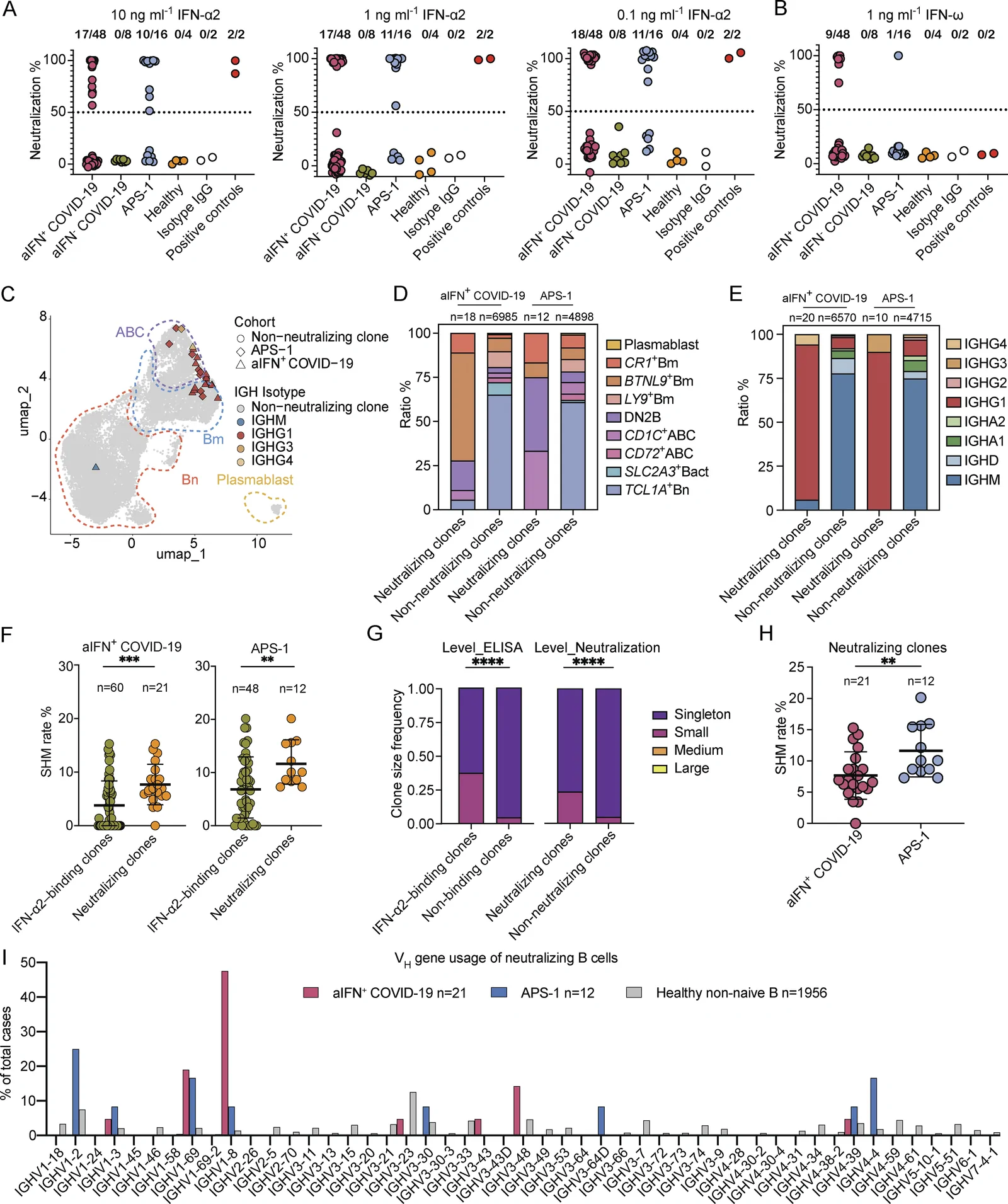
**Molecular profiling of neutralizing clones. (A and B)** Neutralization activity of purified mAbs against IFN-α2 (A) and IFN-ω (B). The cutoff for neutralization was defined as ≥50% neutralizing activity. Representative data from two independent experiments are shown in panels A and B. Each data point represents the mean of duplicate measurements. **(C)** UMAP visualization of confirmed neutralizing clones, defined as B cell clones encoding recombinant antibodies that inhibited IFN-α2 and/or IFN-ω activity in a cell-based neutralization assay, colored by cohort and IGH isotype. **(D and E)** Comparison of B cell subcluster distribution (D) and immunoglobulin isotypes (E) between neutralizing clones and non-neutralizing clones isolated from individuals with aIFN^+^ COVID-19 and APS-1. **(F)** Comparison of SHM frequencies among IFN-α2–binding and neutralizing clones derived from aIFN^+^ COVID-19 and APS-1 patients. **(G)** Comparison of clone-size frequency among IFN-α2–binding and non-binding clones (validated by ELISA), and neutralizing and non-neutralizing clones (validated by neutralization assay). **(H)** Comparison of SHM frequencies among neutralizing clones from individuals with aIFN^+^ COVID-19 and APS-1. **(I)** IGHV gene usage among neutralizing clones from individuals with aIFN^+^ COVID-19 or APS-1, compared with the repertoire of non-naive B cells from healthy donors. Statistical significance for panels F and H was determined using the Wilcoxon rank-sum test and for panel G using Fisher’s exact test; **P < 0.01, ***P < 0.001, and ****P < 0.0001.

21 high-confidence candidates, together with 6 exploratory clones from the initial antibody expression supernatant screen, were subsequently produced at a larger scale and characterized for their neutralizing activities against IFN-α2 and IFN-ω. These were analyzed together with 49 purified mAbs selected directly from the AlphaFold 3-based screening. 27 antibodies neutralized IFN-α2 at stimulation concentrations of 10 ng ml^−1^ (Fig. 5 A and Table 1), including 17 antibodies from aIFN^+^ severe COVID-19 patients and 10 from APS-1 patients. At lower IFN-α2 stimulation concentrations (1 ng ml^−1^ and 0.1 ng ml^−1^), one additional neutralizing antibody was identified in each aIFN^+^ disease cohort, resulting in a total of 29 neutralizing antibodies identified. Notably, among the 57 clones with an ipTM value ≥0.8 that were tested for neutralization, 26 (45.6%) were validated as IFN-α2–neutralizing antibodies (Fig. 4 C).

**Table 1.** Summary of IFN-α2– and IFN-ω–neutralizing autoAbs and their corresponding B cell identities

mAb	Cohort	Donor	Clone frequency	B cell subtype	Heavy chain				Light chain				Neutralization %			
					v_call	j_call	c_call	SHM %	v_call	j_call	c_call	SHM %	10 ng ml^−1^ IFN-α2	1 ng ml^−1^ IFN-α2	0.1 ng ml^−1^ IFN-α2	1 ng ml^−1^ IFN-ω
CTP03	aIFN^+^COVID-19	COV2	3	*BTNL9* ^+^Bm	IGHV1-69	IGHJ4	IGHG1	8.7	IGKV3-20	IGKJ1	IGKC	8.2	100.1	100.1	100.6	98.8
CTP32	aIFN^+^COVID-19	COV6C	1	*CR1* ^+^Bm	IGHV1-8	IGHJ4	IGHG1	5.6	IGKV1-33	IGKJ4	IGKC	6.8	69.5	96.3	94.3	100.0
CTP57	aIFN^+^COVID-19	COV5B	1	*BTNL9* ^+^Bm	IGHV1-8	IGHJ6	IGHG1	6.9	IGKV3-20	IGKJ3	IGKC	5.3	99.7	100.0	98.5	9.2
CTP71	aIFN^+^COVID-19	COV5B	N/A	N/A	IGHV1-8	IGHJ4	IGHG1	7.6	IGKV1-33	IGKJ4	IGKC	6.1	100.1	100.0	97.6	99.6
CTP73	aIFN^+^COVID-19	COV5B	1	*BTNL9* ^+^Bm	IGHV1-8	IGHJ4	IGHG1	4.9	IGKV1-9	IGKJ3	IGKC	2.2	98.5	99.8	100.2	92.3
CTP86	aIFN^+^COVID-19	COV5B	1	*BTNL9* ^+^Bm	IGHV1-3	IGHJ4	IGHG1	13.5	IGKV3-20	IGKJ2	IGKC	5.7	100.1	100.1	101.3	9.0
CTP90	aIFN^+^COVID-19	COV5B	1	*CD1C* ^+^ABC	IGHV4-39	IGHJ4	IGHG1	3.4	IGKV3-15	IGKJ4	IGKC	2.5	4.9	10.5	16.5	97.0
CTP92	aIFN^+^COVID-19	COV5B	1	*BTNL9* ^+^Bm	IGHV1-8	IGHJ5	IGHG1	5.6	IGKV1-33	IGKJ3	IGKC	3.6	100.1	99.9	102.0	98.0
CTP97	aIFN^+^COVID-19	COV5B	1	*BTNL9* ^+^Bm	IGHV1-8	IGHJ6	IGHG1	3.5	IGKV1D-39	IGKJ1	IGKC	2.9	99.3	99.8	103.7	10.1
CTP114	aIFN^+^COVID-19	COV5B	N/A	N/A	IGHV3-48	IGHJ5	IGHG1	15.3	IGLV6-57	IGLJ3	IGLC3	7.2	74.9	97.4	105.3	11.7
CTP118	aIFN^+^COVID-19	COV5B	1	DN2B	IGHV3-43	IGHJ4	IGHG1	14.2	IGLV6-57	IGLJ3	IGLC3	5.8	93.8	99.8	103.7	12.1
CTP119	aIFN^+^COVID-19	COV5B	1	*BTNL9* ^+^Bm	IGHV1-69	IGHJ6	IGHG1	12.2	IGKV1-5	IGKJ2	IGKC	18.4	56.8	92.8	100.1	6.0
CTP145	aIFN^+^COVID-19	COV5B	1	DN2B	IGHV3-23	IGHJ4	IGHG1	5.9	IGLV1-44	IGLJ3	IGLC3	6.3	81.4	96.8	100.7	7.0
CTP165	aIFN^+^COVID-19	COV5B	1	*BTNL9* ^+^Bm	IGHV1-8	IGHJ6	IGHG1	6.3	IGKV1-33	IGKJ4	IGKC	2.2	98.4	100.2	100.7	75.9
CTP171	aIFN^+^COVID-19	COV5B	1	*BTNL9* ^+^Bm	IGHV1-8	IGHJ3	IGHG1	6.3	IGKV1-33	IGKJ4	IGKC	8.6	100.2	100.3	102.6	100.1
CTP182	aIFN^+^COVID-19	COV5B	1	*TCL1A* ^+^Bn	IGHV3-48	IGHJ4	IGHM	0.0	IGKV3-20	IGKJ1	IGKC	0.0	−0.7	31.0	98.0	3.4
CTP190	aIFN^+^COVID-19	COV5B	1	*CR1* ^+^Bm	IGHV1-8	IGHJ6	IGHG1	6.6	IGKV1-33	IGKJ4	IGKC	5.4	100.2	100.2	103.6	97.3
CTP220	aIFN^+^COVID-19	COV5B	1	DN2B	IGHV1-8	IGHJ6	IGHG4	6.9	IGKV1D-39	IGKJ1	IGKC	8.2	95.4	99.8	103.4	21.6
CTP227	aIFN^+^COVID-19	COV5B	N/A	N/A	IGHV3-48	IGHJ5	IGHG1	10.4	IGLV6-57	IGLJ2	IGLC3	3.1	67.4	96.3	98.2	12.6
AH212	APS-1	AP1	1	*CD1C* ^+^ABC	IGHV1-2	IGHJ4	IGHG1	20.1	IGLV2-23	IGLJ1	IGLC1	8.3	72.0	96.2	107.0	11.0
ATP01	APS-1	AP1	2	*CD1C* ^+^ABC	IGHV4-4	IGHJ4	IGHG1	7.3	IGLV3-1	IGLJ3	IGLC2	3.2	51.5	91.6	90.2	7.2
ATP03	APS-1	AP1	1	*CR1* ^+^Bm	IGHV1-3	IGHJ4	IGHG1	8.7	IGKV3-20	IGKJ1	IGKC	5.0	13.2	56.2	78.1	7.8
ATP05	APS-1	AP1	1	*CR1* ^+^Bm	IGHV3-64D	IGHJ3	IGHG1	8.7	IGKV1-16	IGKJ2	IGKC	5.7	65.1	94.9	106.5	9.3
ATP10	APS-1	AP1B	1	DN2B	IGHV1-69	IGHJ4	IGHG1	8.3	IGKV1D-39	IGKJ1	IGKC	3.2	99.8	100.5	101.5	11.8
ATP11	APS-1	AP1B	1	*BTNL9* ^+^Bm	IGHV1-8	IGHJ4	IGHG1	10.1	IGKV1-33	IGKJ5	IGKC	6.5	97.1	99.9	99.1	100.1
ATP13	APS-1	AP3	1	*CD1C* ^+^ABC	IGHV1-69	IGHJ4	IGHG1	16.0	IGKV1D-39	IGKJ2	IGKC	14.7	99.3	100.2	101.9	10.9
ATP16	APS-1	AP5	2	DN2B	IGHV1-2	IGHJ4	IGHG3	15.6	IGLV3-1	IGLJ2	IGLC2	8.6	100.0	100.1	102.7	6.8
ATP19	APS-1	AP5	1	DN2B	IGHV3-30	IGHJ4	N/A	10.1	IGKV1-5	IGKJ1	IGKC	6.5	98.6	100.0	103.0	10.3
ATP31	APS-1	AP5	2	DN2B	IGHV1-2	IGHJ4	N/A	12.2	IGLV3-1	IGLJ2	IGLC2	8.6	99.4	100.0	106.1	11.2
ATP42	APS-1	AP4	1	DN2B	IGHV4-39	IGHJ5	IGHG1	15.4	IGKV3-11	IGKJ4	IGKC	5.7	100.0	100.1	107.8	12.2

ATP16 and ATP31 share the same clone group, but have distinct BCR sequences.

Among all IFN-α2–neutralizing mAbs, eight (CTP03, CTP32, CTP71, CTP73, CTP92, CTP165, CTP171, and CTP190) from aIFN^+^ severe COVID-19 patients and one from an APS-1 patient also cross-neutralized IFN-ω at a stimulation concentration of 1 ng ml^−1^ (Fig. 5 B). In addition, one mAb (CTP90) from an aIFN^+^ severe COVID-19 patient neutralized IFN-ω but not IFN-α2. From this point onward, neutralizing clones were defined as B cells encoding recombinant mAbs that inhibited IFN-α2 and/or IFN-ω activity in a cell-based neutralization assay at the indicated stimulation concentrations and predefined cutoff (≥50%). Non-neutralizing clones comprised recombinant mAbs that did not exhibit IFN-neutralizing activity, as well as BCRs derived from IFN-α2-tetramer– and monomer–negative B cells that were not subjected to functional testing.

Next, 33 B cells carrying 30 distinct neutralizing BCRs described above, including IFN-α2–neutralizing clones and the IFN-ω–neutralizing clone CTP90, were further investigated. Most neutralizing clones originated from mature B cell compartments, including *BTNL9*^*+*^Bm, *CR1*^*+*^Bm, DN2B, and *CD1C*^+^ABC subclusters, which together accounted for 29 of the 30 neutralizing B cell clones for which B cell subtype assignment was possible (96.7%) (Fig. 5 C and Table 1).

Neutralizing clones derived from aIFN^+^ severe COVID-19 patients were predominantly mapped to the *BTNL9*^+^ Bm subcluster (61.1 vs. 7.7% of non-neutralizing clones), whereas neutralizing clones derived from APS-1 patients were preferentially enriched in the DN2B (41.7 vs. 6.0%) and *CD1C*^+^ ABC (33.3 vs. 6.5%) subclusters (Fig. 5 D). Notably, one weak IFN-α2–neutralizing clone from an aIFN^+^ severe COVID-19 patient was derived from a *TCL1A*^+^ Bn lacking SHMs (Fig. 5 A and Table 1), indicating the presence of a naive/germline BCR repertoire capable of recognizing, and in rare cases neutralizing, physiological levels of IFN-α (Rodero et al., 2017).

Consistent with previous reports showing that IgG antibodies are the dominant mediators of type I IFN neutralization (Bastard et al., 2020), neutralizing clones were enriched for IgG subclasses, predominantly IGHG1 (90 vs. 6.4% of non-neutralizing clones in aIFN^+^ COVID-19; 90 vs. 9.0% in APS-1), with smaller proportions of IGHG3 and IGHG4 (Fig. 5 E).

Furthermore, neutralizing clones showed increased SHM levels compared with IFN-α2–binding clones (Fig. 5 F). Neutralizing and IFN-α2–binding clones also exhibited greater clonal expansion than non-neutralizing and non-binding clones, respectively (Fig. 5 G). Notably, neutralizing clones from aIFN^+^ severe COVID-19 patients showed lower overall SHM frequencies than those from APS-1 patients (Fig. 5 H), as previously reported (Fournier et al., 2026), likely reflecting the shorter affinity maturation period during acute viral infection compared with the prolonged maturation of type I IFN-α2–neutralizing B cell responses in APS-1.

Type I IFN–neutralizing clones exhibited a more convergent pattern of *IGHV* gene usage than the IFN-α2–binding repertoire. *IGHV1–8*, which has been associated with autoimmune responses (Lomakin et al., 2014), was the most frequently used gene in aIFN^+^ severe COVID-19 patients (Fig. 5 I), increasing from ∼17% among IFN-α2–binding clones to nearly 50% among neutralizing clones. The light chain gene *IGKV1–33* was overrepresented in these patients and was frequently paired with *IGHV1–8* (Table 1). *IGHV1–2* was the most frequently used gene in APS-1 patients. *IGHV1-69* was the second most frequently used gene for both patient groups, consistent with its reported enrichment in both autoimmune and antiviral immune responses (Avnir et al., 2017). Additionally, modestly biased usage of *IGHV3–48* was observed in aIFN^*+*^ severe COVID-19 patients, whereas *IGHV4–4* and *IGHV3–64D* were preferentially used in APS-1 patients.

To assess the antigenic specificity of IFN-α2–neutralizing mAbs and test whether they might derive from SARS-CoV-2- or autoantigen-cross-reactive clones, we evaluated their reactivity against SARS-CoV-2 antigens (receptor-binding domain and N protein) by ELISA and screened for autoreactivity using a HEp-2 cell-based indirect immunofluorescence assay (Wardemann et al., 2003). None showed detectable binding to viral antigens or HEp-2 autoantigens, indicating specificity for IFN-α2.

In summary, B cells carrying IFN-α2–neutralizing BCRs were identified almost exclusively within non-naive B cell compartments, with enrichment in several B cell subclusters. Neutralizing clones from aIFN^+^ severe COVID-19 patients were predominantly derived from *BTNL9*^+^ Bm, whereas those from APS-1 patients were enriched within the extrafollicular *CD1C*^+^ ABC/DN2B pathway. These differences, together with differences in SHM levels and partially overlapping *IGHV* gene usage, suggest that IFN-α2–neutralizing responses develop through different B cell maturation pathways in the two disease settings. Nevertheless, neutralizing clones from both cohorts exhibited increased SHM levels compared with IFN-α2–binding clones, indicating that IFN-α2–neutralizing activity is acquired through antigen-driven selection and affinity maturation from a diverse physiological B cell repertoire, despite differences in cellular origins.

### IFN-α2–neutralizing antibodies segregate into distinct competition groups and structural subclasses

**Figure 6.**
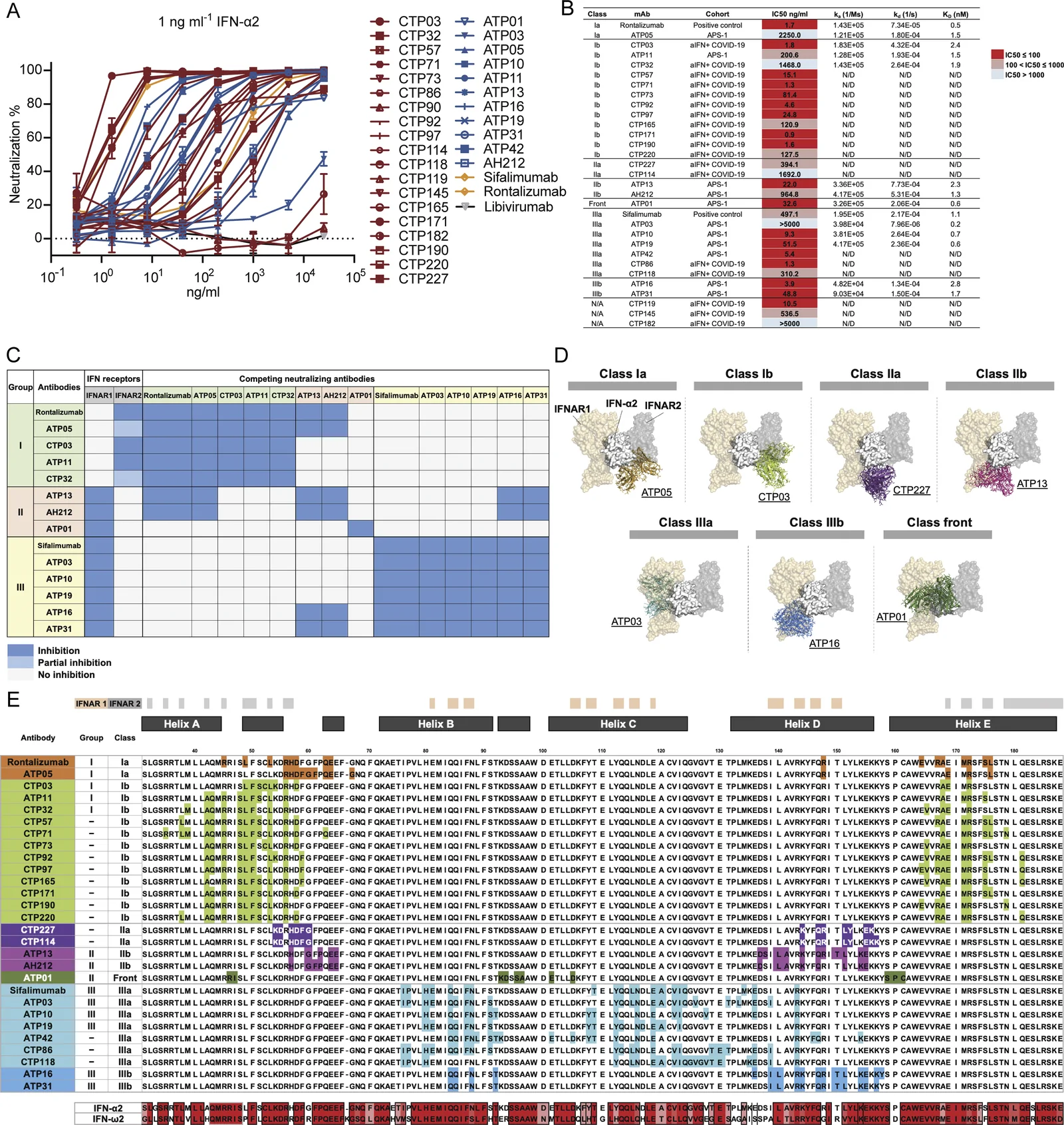
**Functional** **and kinetic characterization of IFN-α2–neutralizing mAbs with predicted structural binding classes. (A)** Dose-dependent neutralization of 1 ng ml^−1^ IFN-α2 by the indicated mAbs. Rontalizumab and sifalimumab served as positive-control IgG antibodies, whereas Libivirumab served as the negative control IgG antibody. Representative data from two to three independent experiments are shown. Symbols show mean neutralization, with error bars indicating variability between replicates. **(B)** Kinetic parameters and affinity constants of representative mAbs, grouped by competition group. IC_50_ values are shown alongside *k*_*a*_, *k*_*d*_, and *K*_*D*_. **(C)** Competition matrix showing inhibition of IFN-α2 binding to IFNAR1 or IFNAR2 and pairwise competition among the indicated antibodies. Dark blue, light blue, and gray denote inhibition, partial inhibition, and no inhibition, respectively. Antibodies were assigned to three principal competition groups. Selected measurements were repeated when necessary to confirm data quality and consistency. **(D)** Competition-supported structural models illustrating representative antibody-binding modes relative to the IFN-α2–IFNAR1–IFNAR2 complex. IFNAR1, IFN-α2, and IFNAR2 are shown in beige, white, and gray, respectively; antibody footprints are colored according to predicted structural binding subclass (Ia, Ib, IIa, IIb, IIIa, IIIb, or front). **(E)** Sequence-based mapping of antibody footprints onto IFN-α2. mAbs are organized by competition group and structural class, with colored residues indicating the predicted contact positions for each antibody. IFNAR1- and IFNAR2-interacting residues and IFN-α helices A–E are annotated above the alignment; the IFN-α2 and IFN-ω sequences are shown below for reference.

**Figure S4.**
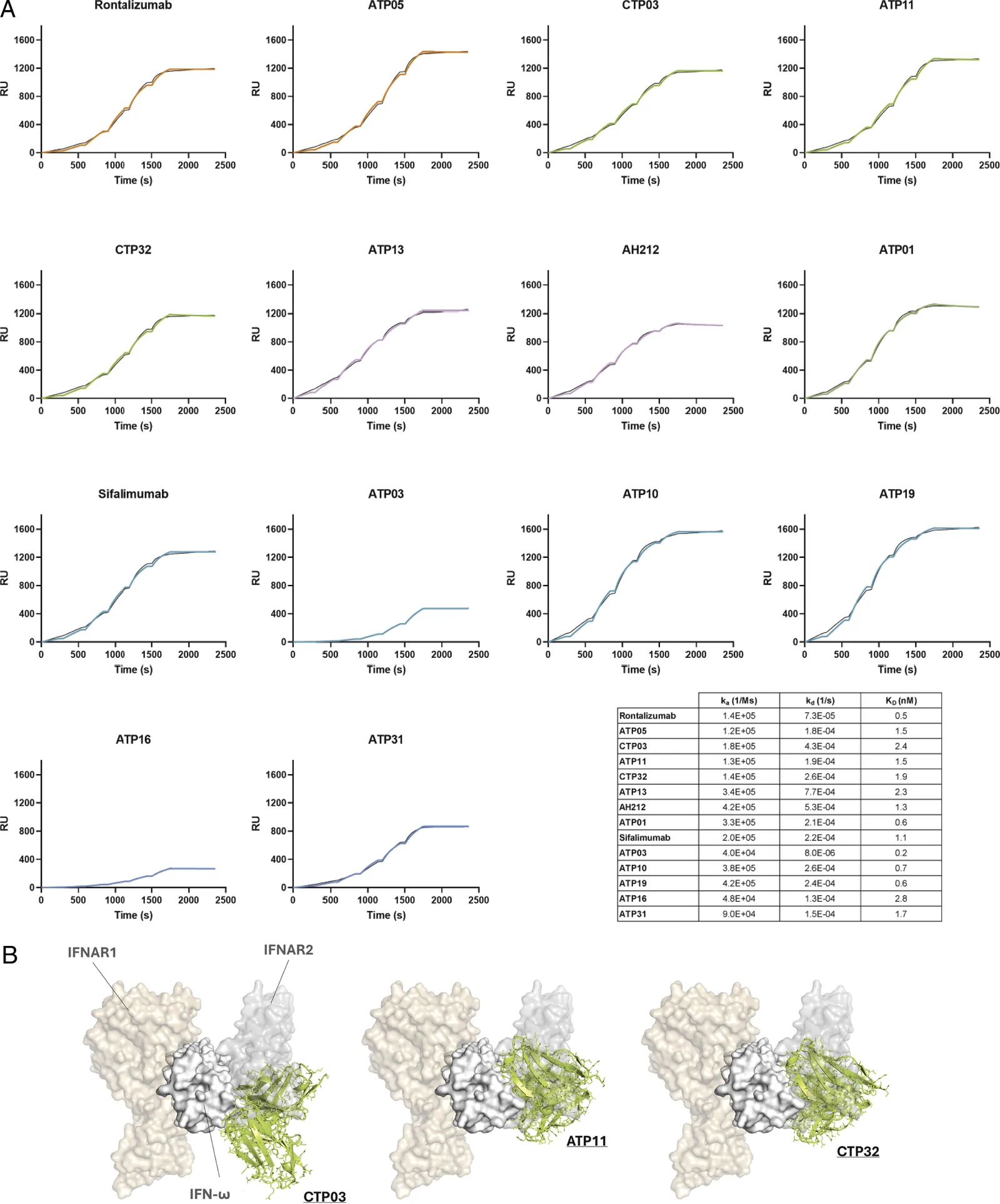
**Related to Fig. 6. (A)** SPR sensorgrams of IFN-α2–neutralizing mAbs injected over immobilized IFN-α2. mAbs were injected at increasing concentrations (0.5–120 nM) using a single-cycle kinetic protocol. Colored lines indicate fitted binding curves, and the corresponding kinetic parameters are summarized in the table. **(B)** Computational docking models of IFN-ω in complex with IFNAR1, IFNAR2, and representative class Ib IFN-α2–neutralizing antibodies (CTP03, ATP11, and CTP32). IFN-ω is shown as a white surface, IFNAR1 and IFNAR2 are shown as surfaces colored ivory and light gray, respectively, and antibodies are shown in green. The models illustrate predicted binding orientations relative to the IFNAR-interacting surface of IFN-ω.

To define the molecular basis of IFN-α2 neutralization, we first profiled the dose-dependent inhibitory activity of isolated mAbs under IFN-α2 stimulation at 1 ng ml^−1^ (Fig. 6, A and B). Titration curves identified 17 mAbs with potent neutralizing activity (IC_50_ ≤100 ng ml^−1^), exhibiting IC_50_ values comparable with or lower than those of the commercial antibodies rontalizumab and sifalimumab (IC_50_ = 1.7 and 497.1 ng ml^−1^, respectively; Fig. 6 B). Seven mAbs displayed moderate neutralizing activity (100 < IC_50_ ≤1,000 ng ml^−1^), whereas an additional five mAbs exhibited weak (IC_50_ >1,000 ng ml^−1^) dose-dependent inhibition of IFN-α2 signaling. The IFN-ω–neutralizing clone CTP90 showed no cross-neutralizing activity against IFN-α2. To assess the contribution of binding kinetics to functional activity, we measured mAb–IFN-α2 interactions by SPR (Fig. 6 B and Fig. S4 A). All tested patient-derived IFN-α2–neutralizing mAbs, as well as the benchmark antibodies rontalizumab and sifalimumab, bound IFN-α2 with high affinity ranging from 0.2 to 2.8 nM (Fig. 6 B). Although affinity contributed to neutralizing activity, it was not sufficient to explain functional potency, highlighting the importance of epitope specificity. We therefore sought to determine whether differences in neutralization potency could be explained by distinct patterns of receptor interference and antigen recognition.

We next applied a two-level classification framework adapted from recent type I IFN epitope-binning and footprint-mapping approaches (Fournier et al., 2026; Groen et al., 2025). First, a representative antibody set was grouped experimentally using a hierarchical competition strategy. Antibodies were initially categorized according to competition with IFNAR1 or IFNAR2, then positioned relative to the benchmark antibodies rontalizumab and sifalimumab, and finally refined through competition among patient-derived mAbs to define the final experimental groups (Fig. 6 C). This experimental classification distinguished mAbs that interfered primarily with IFNAR2 binding and competed with rontalizumab (group I), mAbs that interfered with IFNAR1 binding but showed little or no competition with either rontalizumab or sifalimumab (group II), and mAbs that interfered with IFNAR1 binding and competed with sifalimumab (group III).

Structural modeling and sequence analysis further resolved the experimentally defined competition groups into predicted binding classes relative to the IFNAR1- and IFNAR2-interacting surfaces on IFN-α2, enabling assignment of most neutralizing mAbs within this structural framework (Fig. 6, D and E). Group I mAbs occupied classes Ia and Ib epitopes on the IFNAR2-facing surface. Group II mAbs displayed distinct IFNAR1-associated binding modes that were structurally separated from the benchmark-antibody footprints and were assigned to classes IIa and IIb. Group III mAbs mapped predominantly to class IIIa or IIIb footprints associated with the IFNAR1-interacting surface. ATP01 was assigned to the structurally distinct front class, which was spatially separated from the major group I–III epitopes and predicted to interfere less extensively with receptor binding than the major receptor-blocking subclasses. Three mAbs (CTP119, CTP145, and CTP182) remained unclassified because neither the competition data nor the AlphaFold 3-derived models provided sufficient confidence for assignment (ipTM <0.8). Overall, antibodies with similar competition profiles generally mapped to related structural footprints, whereas structural subclassification provided additional resolution within the experimentally defined groups.

Representative structural models demonstrated that potent neutralizing mAbs were distributed across multiple subclasses, including class Ib, class IIb, and classes IIIa–IIIb, indicating that strong neutralization can arise through engagement of distinct receptor-interfering surfaces rather than a single dominant epitope. COVID-19-derived neutralizing mAbs were preferentially assigned to group I, particularly class Ib, whereas APS-1–derived mAbs were distributed across multiple structural subclasses, consistent with a broader diversity of IFN-α2 recognition modes.

**Figure 7.**
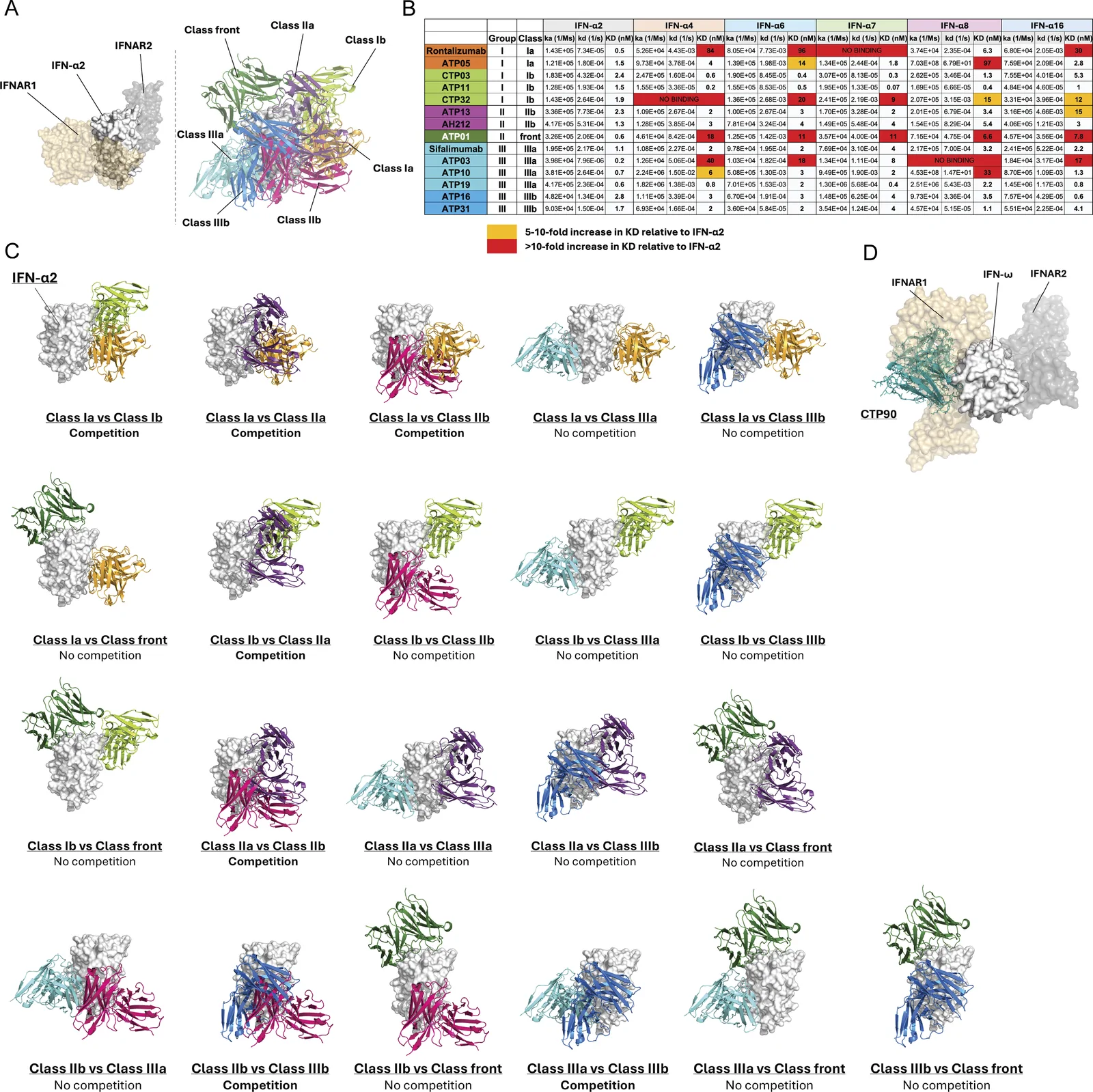
**Structural classification, cross-reactivity, and competition profiles of type I IFN–neutralizing mAbs. (A)** Overview of the seven competition-supported antibody-binding classes identified around IFN-α2: Ia, Ib, IIa, IIb, IIIa, IIIb, and front. IFN-α2 is shown as a gray surface, and representative mAbs are shown as colored ribbons. **(B)** SPR-derived binding kinetics of representative mAbs to IFN-α2, IFN-α4, IFN-α6, IFN-α7, IFN-α8, and IFN-α16. *k*_*a*_, *k*_*d*_, and *K*_D_ are reported. Yellow and red indicate 5–10-fold and >10-fold increases in *K*_D_, respectively, relative to the corresponding value for IFN-α2; “no binding” indicates no measurable binding was detected. SPR measurements were performed using single-cycle concentration-series kinetics analyses, as described in Materials and methods. Selected measurements were repeated when necessary to confirm data quality and consistency. **(C)** Pairwise structural comparison of representative antibody classes bound to IFN-α2. Superimposed models illustrate whether antibodies from different classes have overlapping binding sites and are therefore predicted to compete. Each pair is designated as “competition” or “no competition.” **(D)** Structural model of CTP90 bound to the IFN-ω–IFNAR1–IFNAR2 signaling complex. IFNAR1, IFN-ω, and IFNAR2 are shown as beige, white, and gray surfaces, respectively, whereas CTP90 is shown as a teal ribbon.

Superposition of representative structures revealed that subclasses Ia, Ib, IIa, IIb, IIIa, IIIb, and the front class occupy distinct but partially overlapping regions of the IFN-α2 surface (Fig. 7 A). We next evaluated binding to selected IFN-α subtypes that differ from IFN-α2 at defined amino acid positions, providing an experimental test of residue dependence within the predicted binding classes (Fig. 7 B). Notably, the tested representative mAbs assigned to class IIb and class IIIb consistently retained binding across the entire panel of tested IFN-α subtypes, suggesting that these subclasses target relatively conserved cytokine surfaces. In contrast, classes Ib and IIIa exhibited greater functional heterogeneity in subtype recognition, suggesting greater dependence on residues that vary among IFN-α family members. Although some mAbs within these classes were broadly cross-reactive, these classes also included antibodies with restricted subtype-binding profiles, such as CTP32 (class Ib) and ATP03 and ATP10 (class IIIa), which displayed reduced or absent binding to one or more IFN-α family members. Similarly, the front class mAb ATP01 displayed a restricted binding profile. These findings indicate that mAbs assigned to the same broad structural region can nevertheless differ in their dependence on particular IFN-α amino acid residues that vary among subtypes. Overall, these subtype-binding patterns provide orthogonal experimental support for the proposed structural classification and further demonstrate that certain subclasses, particularly IIb and IIIb, exhibit broader cross-reactivity across IFN-α family members than others.

Having experimentally assessed the predicted binding classes using subtype recognition patterns, we next compared representative antibody footprints to evaluate potential competition relationships among the structural subclasses (Fig. 7 C). Predicted overlap was observed among several subclass pairs, consistent with competition for partially shared IFN-α2 surfaces, whereas other subclass combinations showed little or no overlap and were therefore compatible with simultaneous binding (Fig. 7 C). These relationships provided a structural rationale for the experimentally observed competition patterns and further refined the subclass framework.

One notable exception was CTP90, which was assigned to structural subclass IIIa but neutralized IFN-ω rather than IFN-α2. To investigate this discrepancy, we modeled the interaction between CTP90 and IFN-ω (Fig. 7 D). The predicted binding mode localized to the IFNAR1-facing surface of IFN-ω and was highly similar to that predicted for other class III mAbs despite its distinct cytokine specificity. Related IFN-ω docking models generated for representative class Ib mAbs (CTP03, ATP11, and CTP32) are shown in Fig. S4 B. Together, these models illustrate that antibodies occupying similar structural regions can nevertheless display distinct cytokine-recognition profiles. This finding suggests that local sequence and surface features, in addition to overall binding topology, contribute to cytokine specificity.

Together, these findings demonstrate that IFN-α2–neutralizing antibodies segregate into three experimentally defined competition groups (groups I–III) and seven predicted structural subclasses (Ia, Ib, IIa, IIb, IIIa, IIIb, and a front class) that correspond to distinct receptor-interfering binding modes. The concordance between competition profiling, structural modeling, subtype-binding analyses, and the CTP90–IFN-ω model provides convergent support for this classification framework while maintaining a clear distinction between direct experimental evidence and model-derived structural interpretation.

## Discussion

The increasing recognition of type I IFN autoAbs as determinants of severe viral disease raises several fundamental questions, including whether these autoAbs are induced by viral infection or pre-exist in the host, and which cellular and molecular pathways are associated with type I IFN autoreactive B cells (Andreakos et al., 2025). While recent studies have linked ABC populations to the overall autoAb production in COVID-19 patients (Yuan et al., 2026) and characterized the repertoire, affinity maturation, and structural features of type I IFN autoAbs (Fournier et al., 2026; Groen et al., 2025), our findings directly connect functionally validated IFN-reactive and IFN-neutralizing autoAbs to specific B cell states and maturation trajectories at single-cell resolution. We show that IFN-α2–binding clones are present within both naive and antigen-experienced B cell compartments and can be identified in aIFN^−^ patients and healthy controls. In contrast, BCRs encoding antibodies with type I IFN–neutralizing activity were predominantly detected within selected Bm and ABC subclusters only in patients with aIFN^+^ severe COVID-19 or the monogenic autoimmune disease APS-1. These neutralizing antibodies were characterized by increased SHM, consistent with affinity maturation and functional selection of IFN-reactive clones. The preferential emergence of pathogenic antibodies from affinity-matured B cells suggests a model in which physiological autoreactivity is converted into pathogenic humoral immunity through disease context-specific B cell maturation pathways. Despite distinct cellular origins, the resulting neutralizing antibodies converged on three major functional epitope groups encompassing seven structural epitope classes on IFN-α2, collectively disrupting IFNAR engagement and downstream signaling.

By integrating scRNA-seq, paired scBCR-seq analyses of tetramer-positive B cells and recombinant antibody production, the neutralizing clones were characterized at single-cell transcriptomic resolution in disease settings. In aIFN^+^ severe COVID-19 patients, the majority of neutralizing clones originated from memory B cell lineages, particularly *BTNL9*^+^ Bm, which were characterized by transcriptional signatures of cellular stress and IFN-γ response. In addition, the identification of one naive-derived BCR from this cohort that neutralized IFN-α2 at physiological concentrations suggests that Bns can encode BCRs capable of recognizing and, in rare cases, neutralizing IFN-α2. In contrast, IFN-α2–neutralizing antibodies from APS-1 patients were predominantly derived from DN2B and *CD1C*^*+*^ ABC subsets, suggesting that these responses arise through an extrafollicular B cell maturation pathway distinct from that observed in aIFN^+^ severe COVID-19.

The predominance of IFN-neutralizing clones within affinity-matured antigen-experienced compartments further suggests that, in severe COVID-19, these responses may arise at least in part from pre-existing antigen-experienced clones, rather than being generated *de novo* during acute infection. Nevertheless, neutralizing antibodies from aIFN^+^ severe COVID-19 patients carried fewer SHMs than those from APS-1 patients, consistent with a shorter period of affinity maturation than that associated with the chronic loss of immune tolerance in APS-1. Despite these differences in cellular origin, transcriptomic profiling of tetramer-enriched B cell populations revealed shared enrichment of BCR signaling, antigen presentation, oxidative stress, and IFN-associated transcriptional programs, including upregulation of genes such as *IFITM2*, *IFITM1*, and *IRF2*. These analyses should, however, be interpreted as describing the broader tetramer-enriched compartment that provides context for the functionally validated IFN-reactive and IFN-neutralizing clones characterized in this study.

Both aIFN^+^ and aIFN^−^ COVID-19 patient groups exhibited antibodies against a broader range of autoantigens and cytokines compared with healthy individuals, suggesting that impaired B cell tolerance can coexist with the presence of type I IFN autoAbs. One possible explanation is that virus-induced inflammatory states may promote transient breaches in B cell tolerance (Castleman et al., 2022). Alternatively, alterations in B cell tolerance may have predated COVID-19 by several years, potentially reflecting age-associated erosion of self-tolerance. Consistent with this view, the longitudinal Swiss HIV Cohort Study detected pre-existing autoAbs against autoimmune disease-associated antigens in individuals who subsequently developed anti-type I IFN autoAbs following viral infection or increased IFN exposure (Fernbach et al., 2024). This interpretation is consistent with the median age of 61.5 years in our aIFN^+^ COVID-19 cohort and with the established association between aging, thymic involution, and increased susceptibility to autoimmune disease (Chang et al., 2025).

APS-1 serves as a positive-control disease context in which type I IFN–neutralizing autoAbs develop at a young age and persist. Although APS-1 pathogenesis is clearly attributed to *AIRE* deficiency, the pathogenic effector B cell populations responsible for autoAb production have rarely been systematically investigated. In this study, we confirmed previous observations that APS-1 patients exhibit a B cell compartment skewed toward more mature B cell lineages (Hetemäki et al., 2024). In addition, we found that B cell clones expressing type I IFN–neutralizing BCRs in this cohort exhibited transcriptional profiles that closely resembled ABC signatures reported across multiple autoimmune and chronic infectious conditions, including SLE, Sjögren’s syndrome, rheumatoid arthritis, malaria, and HIV infection (Imabayashi et al., 2025; Jenks et al., 2020). Collectively, these features are consistent with long-term inflammation-driven B cell activation accompanied by transcriptional programs associated with BCR signaling, IFN-γ, and type I IFN responses. In line with this interpretation, a recent prospective clinical study reported excessive systemic IFN-γ activity in APS-1 patients and demonstrated promising therapeutic responses to JAK inhibition with ruxolitinib in five treated individuals (Oikonomou et al., 2024).

Furthermore, our antibody-classification analysis places COVID-19- and APS-1-derived anti-type I IFN mAbs within a competition- and structure-guided framework broadly consistent with that recently described by Fournier et al. and related IFN-I footprint analyses by Groen et al. Competition SPR analyses and structural modeling identified three major functional epitope groups and seven predicted structural epitope classes. Although the principal receptor-interfering binding modes were shared across disease settings, COVID-19-derived neutralizing antibodies were enriched within the group I/class Ib subclass, whereas APS-1-derived antibodies were distributed across multiple functional groups and structural classes, suggesting greater epitope diversity in APS-1. These findings further emphasize the importance of antibody binding mode and receptor interference, rather than affinity alone, as determinants of type I IFN neutralization. Because structural classes were assigned using competition-supported modeling, future crystallographic or cryo-EM studies will be required to validate individual subclass assignments and atomic contacts at higher resolution.

Limitations of our study should be acknowledged. Access to cells from aIFN^+^ severe COVID-19 cases is limited, and antigen-specific B cells are exceedingly rare. Therefore, we prioritized deep transcriptomic and molecular analyses for each individual case rather than a large cohort size. In addition, pre-pandemic longitudinal sampling will be required to determine whether Bm-derived neutralizing antibodies and their corresponding B cell clones pre-exist before SARS-CoV-2 infection and/or subsequently expand during disease.

In summary, our integrated single-cell transcriptomic, BCR clonal, and functional antibody analyses define the immune context, B cell states, and cellular pathways through which type I IFN–autoreactive B cells arise in severe aIFN^+^ COVID-19 and APS-1 patients. Based on these data, we propose a model comprising at least two parallel B cell response programs. Within an inflammatory and oxidative stress-enriched milieu, antigen-experienced B cells, including Bm and ABC subsets, are associated with IFN-neutralizing antibody responses and likely represent cellular subsets harboring affinity-matured type I IFN–neutralizing BCRs. In parallel, Bn carrying germline-encoded BCRs, such as those using *IGHV1–8* and *IGHV1–69*, with intrinsic reactivity to type I IFNs, may constitute a reservoir of autoreactive potential that, under permissive conditions, could contribute to the generation of functionally neutralizing antibodies through affinity maturation. These findings provide insight into the generation of type I IFN autoAbs across diverse diseases and advance our understanding of infection-associated alterations in B cell regulation that phenocopy certain forms of monogenic IEI. Moreover, type I IFN autoAbs represent a double-edged sword: while they impair antiviral defense, type I IFN–neutralizing mAbs have demonstrated therapeutic potential in multiple systemic autoimmune diseases, including systemic lupus erythematosus and Sjögren’s syndrome, both of which are characterized by a pronounced type I IFN gene signature (Mathian et al., 2022; Tokuyama et al., 2021; Vinuesa et al., 2023).

## Materials and methods

### Study design

The study aimed to characterize type I IFN–neutralizing autoAbs and define their B cell states, cellular compartments, developmental trajectories, and functional properties in patients with aIFN^+^ severe COVID-19 and APS-1. Sera or plasma from COVID-19 and APS-1 patients were first profiled for reactivity to IFNs, cytokines, and autoantigens. Type I IFN–reactive B cells were then enriched and isolated by monomer- or tetramer-based single-cell flow cytometry sorting and subjected to paired scRNA-seq and scBCR-seq to delineate cellular phenotypes, clonal features, and inferred differentiation trajectories. By combining AlphaFold 3 modeling-based IFN-α2–BCR interaction screening and antibody cloning, we functionally validated binding and neutralizing activities using ELISA and a type I IFN neutralization assay. Functional annotations were integrated with single-cell transcriptomic and BCR data to map neutralizing activity to specific B cell states, isotypes, and lineages. Lead neutralizing antibodies were further characterized for affinity, epitope specificity, and IFN-α2 interactions using SPR and structure-guided docking analyses. Sample sizes for each experiment are indicated in the corresponding figures.

### Human samples

Blood samples were collected between April and December of 2020 under ethical approval Dnr 2020-01558 from nine Swedish patients with severe COVID-19 who had previously tested positive for anti–IFN-α2 autoAbs (Strunz et al., 2024). Serum samples were obtained between 9 and 28 days after symptom onset, with a median of 15 days. From the same cohort, PBMC samples from eight patients were collected at the same sampling point. Three longitudinal PBMC samples were also included in the scRNA-seq and scBCR-seq analyses. Two of the longitudinal PBMC samples were sampled at the second and third time points within 30 days of symptom onset, whereas one was sampled at a second time point on day 214 after symptom onset. Blood samples from three patients with aIFN^−^ severe COVID-19, five patients with APS-1, and eight buffy coats from healthy donors were collected under ethical approvals Dnr 2020-01558 and Dnr 2023-02698-02 at Karolinska University Hospital, Stockholm. An additional eleven blood samples from patients with aIFN^−^ severe COVID-19 were collected under ethical approval P_20200029440 at Fondazione IRCCS Policlinico San Matteo, Italy (Sherina et al., 2021). Blood samples from five additional healthy donors (without prior infection or vaccination history; negative control [NC]) were collected under ethical approval Dnr 2020-01612 at the Karolinska Institutet, Stockholm (Table S1). All participants provided written informed consent. PBMCs were isolated from peripheral blood using density gradient centrifugation and subsequently cryopreserved.

### Customized autoantigen array

Customized autoantigen array chips containing ∼500 features, including cytokines, intracellular antigens, autoimmune disease-associated autoantigens, as well as viral antigens (Behere et al., 2025), were blocked in PBS containing 2% BSA and 0.05% Tween-20 for 90 min and incubated overnight with serum from patients and controls diluted 1:2,000. Serum from a panel of healthy donors served as controls. Arrays were washed in PBS–Tween-20 and probed with Alexa Fluor 647–conjugated goat anti-human IgG (1:2,000; #A-21445; Thermo Fisher Scientific) for 90 min. After washing, arrays were rinsed in deionized water, dried by centrifugation, and scanned. Median fluorescence intensities (MFIs) were extracted, followed by background subtraction and normalized across experiments. At the cohort level, IgG-reactive antigen positivity was defined as a log_2_ fold change ≥3.5 relative to the mean of the NC group and MFI ≥500 arbitrary units (log_10_ MFI ≥2.699). At the individual level, positivity was defined using the same MFI threshold but a more stringent log_2_ fold change cutoff of ≥6.644 to facilitate visualization of individual-level autoreactivity profiles.

### Neutralization assay

The neutralizing activity of anti–IFN-α2 antibodies was evaluated using the HEK-Blue IFN-α/β reporter cell line (Cat. #hkb-ifnab; InvivoGen). These cells produce secreted alkaline phosphatase (SEAP) under the ISG54 promoter upon activation by type I IFNs, allowing colorimetric quantification of IFN activity. Candidate antibodies (10 μg ml^−1^) or serum (1:10 diluted) were incubated with IFN-α2 antigen (10, 1, and 0.1 ng ml^−1^) for 2 h at 37°C, followed by co-culture with HEK-Blue cells, as described previously (Behere et al., 2025). After 18–24 h, SEAP activity was measured at 630 nm using QUANTI-Blue substrate, and neutralization activity was calculated as described below. APS-1 patient serum (1:10) and the commercial anti–IFN-α2 mAbs sifalimumab and rontalizumab (10 μg ml^−1^) were included as positive controls, while healthy donor serum and an anti-SARS-CoV-2 RBD IgG isotype control mAb (KI4) were used as NCs. The neutralization activity was calculated using the following equation: Neutralization activity (%) = 100 × [1 – (test sample OD_630_ – unstimulated OD_630_)/(blank OD_630_ – unstimulated OD_630_]. The cutoff for neutralization was defined as ≥50% neutralizing activity.

### Recombinant IFN-α2 antigen

Two biotinylated IFN-α2 proteins were used for IFN-α2–specific B cell sorting. Non-site-specific biotinylated IFN-α2 was generated by biotinylating recombinant IFN-α2 protein (Cat. No. Z03003; GenScript) using the Biotin (Type A) Fast Conjugation Kit (Abcam) according to the manufacturer’s protocol. Recombinant human IFN-α2 (UniProt accession no. P01563, amino acids 1–188) was expressed in a HEK293 expression system (Sino Biological) with a C-terminal His_6_-tag for purification and an Avi-tag for site-specific biotinylation using BirA biotin-protein ligase. The resulting biotinylated IFN-α2 protein was subsequently used to generate IFN-α2 tetramers by mixing it with streptavidin conjugated to R-phycoerythrin (streptavidin-PE) or allophycocyanin (streptavidin-APC) (BioLegend) at a molar ratio of 4.5:1. Both IFN-α2–PE and IFN-α2–APC tetramers were titrated to concentrations of 5–12 nM for B cell sorting.

### Flow cytometry and single-cell sorting

PBMCs were thawed in pre-warmed RPMI 1640 supplemented with 10% FBS, washed with PBS, and stained with Live/Dead Aqua (Thermo Fisher Scientific) for 15 min at 4°C, followed by Fc receptor blocking (Fc block, BD Biosciences) for 10 min at 4°C. The staining mixture was prepared by combining IFN-α2–PE and IFN-α2–APC tetramers with FITC mouse anti-human CD3, CD14, and CD16 antibodies and PE-Cy7 mouse anti-human CD19 (all from BD Biosciences). Cells were incubated with the antibody mixture for 20 min at 4°C. Meanwhile, each donor sample was labeled with a unique hashtag barcode for demultiplexing during scRNA-seq and scBCR-seq analyses. IFN-α2-tetramer–staining positive B (Live/CD3^-^CD14^−^CD16^−^/CD19^+^/IFN-α2-PE^+^/IFN-α2-APC^+^), nonspecific B cells (Live/CD3^−^CD14^−^CD16^−^/CD19^+^/IFN-α2-PE^−^/IFN-α2-APC^−^), and non-B immune cells (Live/CD3^+^/CD14^+^/CD16^+^/CD19^−^) were single-cell sorted by BD FACSAria III sorter (BD Biosciences). The flow-cytometry sorting was performed at the Biomedicum Flow Cytometry Core facility at Karolinska Institutet.

### scRNA-seq and scBCR-seq library preparation and sequencing

Sorted cell suspensions were loaded onto individual lanes of the Chromium Next GEM Controller (10X Genomics) using the Chromium Next GEM Single-Cell 5′ Reagent Kit v2 (10X Genomics) to generate gel bead-in-emulsions (GEMs). GEMs were subjected to reverse transcription, followed by cDNA amplification using a thermal cycler. Single-cell expression libraries were constructed according to the manufacturer’s protocols. BCR-sequencing libraries were generated using the Chromium Single Cell Human BCR Amplification Kit (10X Genomics), following the manufacturer’s instructions. Amplified full-length cDNA was subsequently used to construct gene expression, V(D)J, and hashtag oligo libraries, according to the manufacturer’s protocols. All libraries were sequenced on either the Illumina NovaSeq 6000 platform or the DNBSEQ-G400 platform.

### scRNA-seq data filtering, quality control, and preprocessing

Raw scRNA-seq datasets were pre-processed using Cell Ranger v7.0.0 (10X Genomics) against the GRCh38 human reference genome to generate gene count matrices. Cells were excluded if they met any of the following criteria: fewer than 500 detected genes, >5,000 detected genes, >40,000 total UMIs, >20% mitochondrial gene content, or <5% ribosomal gene content (Wan et al., 2025). Potential doublets were identified and removed using Scrublet. After quality control, count matrices were normalized using the NormalizeData function in Seurat (v.5.1.0) (Hao et al., 2024), and the log-normalized expression matrix was used for downstream analyses.

### Data integration, dimensionality reduction, and unsupervised clustering

To annotate immune cell populations, the top 3,000 highly variable genes (HVGs) were identified using the FindVariableFeatures function in Seurat. PCA was then performed on the HVG matrix. Harmony (v.1.2.0) was applied to correct for batch effects, and the top 30 Harmony components (Korsunsky et al., 2019) were retained for downstream analyses. Dimensionality reduction and unsupervised clustering were then conducted using Seurat’s default parameters. Each cell cluster was annotated based on canonical marker genes to identify major immune cell types. Clusters expressing signatures of distinct cell lineages were removed to eliminate potential doublets.

B cell clusters were subsequently extracted for downstream analysis. The top 2,000 HVGs were selected, followed by PCA and batch correction using Harmony with default parameters. The top 30 Harmony components were used for unsupervised clustering by building a nearest neighbor graph with a resolution of 1.0. For visualization, dimensionality was further reduced using UMAP via the Seurat RunUMAP function with parameter dims = 1:30 and reduction = “harmony.” Default settings were used for all other parameters unless otherwise specified.

Following the first round of unsupervised clustering, B cell clusters were annotated based on the expression of canonical markers, identifying the major subsets, including naive, Bm, ABCs, and plasmablasts. The second round of the clustering procedure followed the same pipeline, beginning with log-transformed expression matrices, top 2,000 HVG selection, PCA, and batch correction with Harmony.

Cluster detection and visualization were repeated for each major B cell subcluster. Clustering resolution parameters were set as follows: Bn, 0.2; Bm and ABC, 0.7. Resolution values were chosen based on the ROGUE (Liu et al., 2020) statistic to balance cluster granularity and purity. DEGs were identified for each subcluster using the FindAllMarkers function in Seurat. Based on these DEGs, B cell subclusters were annotated according to their unique transcriptional profiles relative to the remaining clusters.

### Scoring cells using gene expression signatures

To characterize the transcriptional features of each B cell subset, gene expression signatures from a reference B cell atlas were applied. Signature scores for each B cell were calculated using the AddModuleScore_UCell function in UCell (v.2.6.2), and the mean score of all cells within each subset was used for comparative analysis.

### Differential gene expression and pathway enrichment analysis

DEGs between selected groups were identified using the rank_genes_groups function in Scanpy (v.1.9.5) (Virshup et al., 2023). DEGs were selected using a BH-adjusted P value <0.05 and |log_2_(fold change)| >0.25.

### Pseudotime analysis

To investigate transcriptional trajectories within B cells, we applied Monocle3 (v.1.0.0) (Cao et al., 2019) to reconstruct differentiation trajectories. To maintain consistency in the UMAP visualization, we inherited key components from the Seurat object, including the original count matrix, metadata, feature set, PCA reduction, and UMAP embeddings, to create a Monocle object. Specifically, the principal graphs were fitted using the learn_graph function. The principal graphs visualized on the UMAP as “skeleton lines” indicate the differentiation trajectories. Based on prior biological knowledge, *TCL1A*^+^Bn was selected as the root of the trajectory. Pseudotime was then calculated using the order_cells function.

### BCR repertoire analysis

Raw scBCR-seq data were assembled and quantified using the Cell Ranger V(D)J pipeline with the GRCh38 reference genome. Only productive BCR sequences were retained, and cells containing >1 productive heavy or light chain were excluded for downstream analyses. Paired scBCR-seq data were integrated with scRNA-seq data based on matched cell barcodes. Germline sequences were reconstructed by CreateGermlines.py from Change-O (v.1.3.0) (Gupta et al., 2015). The SHM rate of each B cell was quantified as the point mutation frequency in the IGHV sequence, using the observedMutations function from the ShazaM package (v.1.2.0) (Gupta et al., 2015) with the following parameters: sequenceColumn = “sequence_alignment”, germlineColumn = “germline_alignment_d_mask”, regionDefinition = NULL, frequency = TRUE, combine = TRUE, and nproc = 1. SHM rates were then classified into three categories: low-SHM (<1%), medium-SHM (1–5%), and high-SHM (>5%). Statistical comparisons of SHM rates between B cell subsets were determined using the Wilcoxon rank-sum test.

Clonal grouping was performed using DefineClones.py from Change-O. Clones were defined within each patient based on identical IGHV and IGHJ gene usage, identical CDR3 length, and a CDR3 sequence distance threshold <0.2 calculated as the Hamming distance normalized by junction length. Each unique clonal group was assigned a clone_id. The clonal size for each clone represents the number of cells belonging to that clonal group. To facilitate downstream comparative analyses, clonal sizes (cloneSize) were categorized into commonly used bins: singleton (size = 1), small (2–5), medium (6–20), and large (21–100).

### AlphaFold 3

Structural modeling of the human IFN-α2 (IFNA2) and antibody complex was performed using the AlphaFold 3 server (https://alphafoldserver.com/) between 2024 and 2025. The amino acid sequence of IFN-α2 (UniProt accession: P01563; residues 24–188) was used:

CDLPQTHSLGSRR​TLMLLAQMRRISLFSCLKDRHDFGFP​QE​EFGNQFQKAETIPVLHEMIQQI​FNLFSTKDSSAAWDETLLD​KFY​TELYQ​QLNDLEACVIQGVGVTET​PLMKEDSILAVRKYFQRI​TLYLKE​KKYSPCAWEVVRAEIMRSFSLSTNLQESLRSKE.

The VH and variable light (VL) chain sequences derived from candidate BCRs were modeled together with the IFN-α2 antigen to predict antibody–antigen complex structures. Binding potential was assessed using the ipTM score, which estimates the accuracy of the predicted interchain spatial arrangement within the complex. Complexes with an ipTM score ≥ 0.8 were considered high-confidence predictions and selected as candidate IFN-α2–binding antibodies.

### Antibody selection and production

mAbs were selected for production based on single-cell BCR repertoire data obtained from patients with severe COVID-19, APS-1, and healthy donors. Individual B cells were clustered into clonal groups and assessed according to clonal frequency, isotype, SHM rate, and annotated B cell subtype.

Predicted VH/VL-IFN-α2 complex models generated by AlphaFold 3 were screened for binding potential based on their ipTM scores and clone size. For BCR sequences shared by multiple B cells, only one representative BCR was selected for antibody production.

A total of 240 clones that met at least one of these criteria were subjected to antibody production and subsequent IFN-α2–binding screening. All recombinant antibodies were expressed as human IgG1 irrespective of their original heavy-chain isotype. The VH and VL chain sequences selected for antibody expression were analyzed using IMGT. Full-length V(D)J region sequences were codon-optimized for mammalian expression and synthesized by Sino Biological. Each VH and VL gene was cloned into individual pcDNA3.4 expression vectors containing the corresponding human constant regions: the IgG1 heavy-chain or either the κ or λ light chain constant region. The resulting plasmids were sequence-verified before use in transfection.

### Antibody production

#### 1-ml-scale antibody production for rapid IFN-α2–binding screening

HEK-293FT cells (0.5 × 10^−6^ per well) were seeded into 24-well plates and incubated overnight at 37°C. The next day, equimolar amounts of heavy- and light-chain plasmids (1 µg total DNA) were transfected using polyethyleneimine (PEI) according to the manufacturer’s protocol. 6 to 8 h after transfection, the medium was replaced with 1 ml of fresh DMEM containing 10% FBS and 1% penicillin-streptomycin. Cells were cultured at 37°C, 5% CO_2_, and 60% humidity. Culture supernatants were harvested 3 days later and stored at −20°C until tested for IFN-α2 binding.

#### 30-ml-scale antibody production and purification

ExpiCHO-S cells were seeded the day before transfection at a density of 3 × 10^−6^ cells ml^−1^. On the day of transfection, paired heavy- and light-chain plasmids (2 μg ml^−1^ total DNA; 1:3 heavy-to-light molar ratio) were mixed and transfected using PEI according to the manufacturer’s instructions. Cells were cultured for 6 days at 37°C, 8% CO_2_, 70% humidity, and 130 rpm. Culture supernatants were collected, and IgG antibodies were purified using Protein G Sepharose 4 Fast Flow (GE Healthcare). The antibody-containing eluates were buffer-exchanged into PBS using Zeba Spin desalting columns (Thermo Fisher Scientific).

### ELISAs

ELISAs were performed to measure the binding capacity of recombinant antibodies to the IFN-α2 antigen. High-binding ELISA plates (#3690; Corning) were coated with 5 μg ml^−1^ Neutravidin (Thermo Fisher Scientific) in PBS at 4°C overnight, followed by conjugation with Avi-tagged recombinant human biotinylated IFN-α2 antigen. HEK293FT culture supernatants and purified mAbs at 1 μg ml^−1^ were diluted in PBS supplemented with 0.1% (vol/vol) BSA and added to the coated wells. Bound antibodies were detected using HRP-conjugated goat anti-human (H+L) secondary antibodies (#A18805; Thermo Fisher Scientific). Color development was achieved using tetramethylbenzidine substrate ( #T0440; Sigma-Aldrich). The reaction was stopped after 10 min with 0.5 M H_2_SO_4_, and absorbance was measured at 450 nm using an ELISA plate reader. Anti–IFN-α2 antibody levels were quantified using an in-house purified human IgG standard (Karolinska University Hospital) with a known concentration.

### SPR

All SPR experiments were performed on a Biacore 8K instrument (Cytiva) at 25°C. The running buffer consisted of 10 mM HEPES (pH 7.4), 150 mM NaCl, 3 mM EDTA, and 0.005% Tween-20.

For kinetic analysis of antibody binding to IFNs, IFN-α2, IFN-α4, IFN-α6, IFN-α7, IFN-α8, and IFN-α16 were individually immobilized on CM5 sensor chips (Cytiva) by standard amine-coupling chemistry. Antibodies were injected at increasing concentrations (0.5, 1.5, 4.4, 13.3, 40, and 120 nM) using a single-cycle kinetics protocol followed by a 10-min dissociation phase. Sensorgrams were reference-subtracted to correct for bulk refractive-index changes and nonspecific binding. Binding kinetics were analyzed using a 1:1 Langmuir interaction model in Biacore Insight Evaluation Software (version 5.0.18.22102), and association rate ($k_{a}$), dissociation rate ($k_{d}$), and equilibrium dissociation constant ($K_{D}$) were determined from the fitted curves.

Competition assays were performed to assess the ability of antibodies to inhibit IFN-α2 binding to its receptor subunits. Human IFNAR1 and IFNAR2 proteins (ACROBiosystems) were immobilized on CM5 sensor chips using standard amine-coupling chemistry. Antibodies (200 nM) were preincubated with IFN-α2 (100 nM), and the resulting complexes were injected over immobilized IFNAR1 or IFNAR2. IFN-α2 alone (100 nM) served as a positive control. Retention of binding indicated that the antibody did not interfere with the interaction between IFN-α2 and the respective receptor subunit, whereas loss of binding indicated competitive inhibition.

Epitope-binning experiments were performed using a sandwich-format competition assay. Primary antibodies were immobilized on CM5 sensor chips by standard amine-coupling chemistry. IFN-α2 (100 nM) was injected to capture antigen on the immobilized antibody, followed immediately by injection of a secondary antibody (50 nM). Binding of the secondary antibody indicated recognition of a nonoverlapping or spatially distinct epitope, whereas absence of binding indicated recognition of overlapping or sterically incompatible epitopes.

### Computational modeling and epitope definition

To define the antibody–antigen–binding interfaces at the amino acid level, the structural models were analyzed using PyMOL (Schrödinger, LLC). Rather than relying solely on a distance-based threshold, binding epitopes were identified by calculating the solvent-accessible surface area (SASA) of residues within the interacting regions. Interface residues were defined based on the change in SASA between the unbound and complexed states. This computational analysis was complemented by detailed visual inspection of the binding footprints in PyMOL to ensure accurate mapping of the interacting residues.

### Statistics

Statistical analysis was performed using Prism (GraphPad v.10.2) or R (v.4.4.2; release 31 Oct 2024). A P value below 0.05 was considered statistically significant.

### Online supplemental material

Fig. S1 shows cytokine and self-antigen autoreactivity profiles at the individual donor level. Fig. S2 shows the gating strategy used for IFN-α2 monomer- and tetramer-based B cell sorting and the distribution of major immune cell subsets across individual donors. Fig. S3 shows transcriptomic features of IFN-α2-tetramer-positive B cells from patients with aIFN^+^ severe COVID-19, aIFN^−^ severe COVID-19, and APS-1. Fig. S4 shows representative SPR sensorgrams of IFN-α2–neutralizing mAbs injected over immobilized IFN-α2 and computational docking models of representative antibodies in complex with IFN-ω. Tables S1 and S2 summarize cohort and donor information. Table S3 summarizes the source data for Fig.1 and Fig. S1. Table S4 shows data underlying Figs. 2, 3, 4, 5, and 6. Table S5 provides a list of key reagents and resources.

## Supplementary Material

Table S1 shows an overview of cohort information.

Table S2 shows patient cohort information.

Table S3 shows summarizes the source data for Fig.1 and Fig. S1.

Table S4 shows data underlying Figs. 2, 3, 4, 5, and 6.

Table S5 shows key reagents and resources.

Table S6 lists Karolinska KI/K COVID-19 Study Group members’ affiliations.

## Data Availability

All data needed to evaluate the conclusions in the paper are present in the paper and/or the Supplementary Materials. The raw scRNA-seq data have been deposited in the Swedish National Data Service: https://doi.org/10.48723/hqay-t924.
