# Supplementary material for: Cellular origin and characterization of autoantibodies against type I IFNs in COVID-19 and APS-1
Source: J Exp Med. 2026 Nov 3;223(12):e20260268. doi: 10.1084/jem.20260268 (PMC13729907; doi:10.1084/jem.20260268)
Supplement: Supplementary file 1 [file jem_20260268_tables1.docx]

Table S1. Overview of cohort information

| **Cohort** | **Number of individuals** | **Type I IFN autoAb status** | **First sample collection, median days post symptom onset** | **Median age** | **Sex ratio (male/female)** |
| --- | --- | --- | --- | --- | --- |
| aIFN^+^ severe COVID-19 | 9 | + | 15 | 61.5 | 75% (6/2*) |
| aIFN^-^ severe COVID-19 | 14 | - | 17.5 | 64.5 | 71% (10/4) |
| aIFN^-^ mild COVID-19 | 4 | - | 24.5 | 57 | 50% (2/2) |
| APS-1 | 5 | + | N/A | 57 | 40% (2/3) |
| Negative controls (NC)^a^ | 5 | - | N/A | 51 | 60% (3/2) |
| Healthy donors^b^ | 12 | - | N/A | 56 | 50% (4/4**) |

* Demographic information was not available for one donor.

** Demographic information was not available for four donors. N/A, not available.

^a^ Samples collected during the early pandemic (2020-2021), from healthy blood donors without history of SARS-CoV-2 infection or vaccination.

^b^ Samples collected from healthy blood donors during late pandemic (2022-2025) when most of the individuals have either been vaccinated or infected.
