# Supplementary material for: Cellular origin and characterization of autoantibodies against type I IFNs in COVID-19 and APS-1
Source: J Exp Med. 2026 Nov 3;223(12):e20260268. doi: 10.1084/jem.20260268 (PMC13729907; doi:10.1084/jem.20260268)
Supplement: Supplementary file 2 [file jem_20260268_tables2.docx]

Table S2. Patient cohort information

| **Cohort** | **Donor ID** | **Sample collection days post symptom onset** | **Type I IFN autoAb status** | **History of COVID-19** | **COVID-19 severity** | **Age** | **Sex** | **Neutralization assay** | **Autoantigen array** | **scRNA-seq** |
| --- | --- | --- | --- | --- | --- | --- | --- | --- | --- | --- |
| aIFN^+^ COVID-19 | COV1 | 9 | + | Yes | severe | 91 | F | yes | yes | yes |
| aIFN^+^ COVID-19 | COV2 | 23 | + | Yes | severe | 51 | M | yes | yes | yes |
| aIFN^+^ COVID-19 | COV3 | N/A | + | Yes | severe | N/A | N/A | no | no | yes |
| aIFN^+^ COVID-19 | COV4 | 28 (1st sampling) | + | Yes | severe | 50 | F | yes | yes | yes |
| aIFN^+^ COVID-19 | COV4 | 214 (2nd sampling) | + | Yes | severe | 50 | F | yes* | yes* | yes |
| aIFN^+^ COVID-19 | COV5 | 16 (1st sampling) | + | Yes | severe | 66 | M | yes | yes | yes |
| aIFN^+^ COVID-19 | COV5 | 23 (2nd sampling) | + | Yes | severe | 66 | M | yes* | yes* | yes |
| aIFN^+^ COVID-19 | COV5 | 117 (3rd sampling) | + | Yes | severe | 66 | M | yes* | yes* | no |
| aIFN^+^ COVID-19 | COV6 | 9 (1st sampling) | + | Yes | severe | 64 | M | yes | yes | yes |
| aIFN^+^ COVID-19 | COV6 | 16 (2nd sampling) | + | Yes | severe | 64 | M | yes* | yes* | yes |
| aIFN^+^ COVID-19 | COV6 | 30 (3rd sampling) | + | Yes | severe | 64 | M | no | no | yes |
| aIFN^+^ COVID-19 | COV7 | 10 | + | Yes | severe | 52 | M | yes | yes | yes |
| aIFN^+^ COVID-19 | COV8 | 17 (1st sampling) | + | Yes | severe | 59 | M | yes | yes | no |
| aIFN^+^ COVID-19 | COV8 | 24 (2nd sampling) | + | Yes | severe | 59 | M | yes* | yes* | no |
| aIFN^+^ COVID-19 | COV9 | 14 | + | Yes | severe | 70 | M | yes | yes | yes |
| aIFN^-^ COVID-19 | COV11 | 18 | - | Yes | severe | 78 | F | yes | yes | no |
| aIFN^-^ COVID-19 | COV12 | 23 | - | Yes | severe | 73 | M | yes | yes | no |
| aIFN^-^ COVID-19 | COV13 | 12 | - | Yes | severe | 78 | M | yes | yes | no |
| aIFN^-^ COVID-19 | COV15 | 21 | - | Yes | severe | 57 | M | yes | yes | yes |
| aIFN^-^ COVID-19 | COV16 | 35 | - | Yes | critical | 55 | F | yes | yes | no |
| aIFN^-^ COVID-19 | COV17 | 19 | - | Yes | severe | 45 | M | yes | yes | no |
| aIFN^-^ COVID-19 | COV18 | 17 | - | Yes | severe | 67 | M | yes | yes | no |
| aIFN^-^ COVID-19 | COV19 | 20 | - | Yes | severe | 57 | M | yes | yes | yes |
| aIFN^-^ COVID-19 | COV20 | 10 | - | Yes | severe | 57 | M | yes | yes | yes |
| aIFN^-^ COVID-19 | COV21 | 15 | - | Yes | severe | 79 | F | yes | yes | yes |
| aIFN^-^ COVID-19 | COV22 | 22 | - | Yes | severe | 73 | F | yes | yes | yes |
| aIFN^-^ COVID-19 | COV23 | 11 | - | Yes | severe | 62 | M | yes | yes | no |
| aIFN^-^ COVID-19 | COV24 | 16 | - | Yes | severe | 67 | M | yes | yes | no |
| aIFN^-^ COVID-19 | COV25 | 11 | - | Yes | severe | 57 | M | yes | yes | no |
| mild COVID-19 | COV26 | 25 | - | Yes | mild | 61 | F | yes | yes | no |
| mild COVID-19 | COV27 | 24 | - | Yes | mild | 53 | M | yes | yes | no |
| mild COVID-19 | COV28 | 31 | - | Yes | mild | 52 | F | yes | yes | no |
| mild COVID-19 | COV29 | 22 | - | Yes | mild | 81 | M | yes | yes | no |
| APS-1 | AP1 | N/A (1st sampling) | + | N/A | N/A | NR** | NR** | yes | yes | yes |
| APS-1 | AP2 | 4 months (1st sampling) | + | Yes | mild | NR** | NR** | yes | yes | yes |
| APS-1 | AP3 | 43 months | + | Yes | hospitalized | NR** | NR** | yes | yes | yes |
| APS-1 | AP4 | 56 months | + | Yes | moderate | NR** | NR** | yes | yes | yes |
| APS-1 | AP5 | 42 months | + | Yes | hospitalized | NR** | NR** | yes | yes | yes |
| APS-1 | AP1B | N/A (2nd sampling) | + | N/A | N/A | NR** | NR** | yes | yes | yes |
| APS-1 | AP2B | 18 months (2nd sampling) | + | Yes | mild | NR** | NR** | yes | yes | yes |
| Negative control | NC1 | N/A | - | No | N/A | 51 | F | yes | yes | yes |
| Negative control | NC2 | N/A | - | No | N/A | 40 | M | yes | yes | no |
| Negative control | NC3 | N/A | - | No | N/A | 70 | M | yes | yes | no |
| Negative control | NC4 | N/A | - | No | N/A | 53 | M | yes | yes | yes |
| Negative control | NC5 | N/A | - | No | N/A | 38 | F | yes | yes | no |
| Healthy donor | HD1 | N/A | - | N/A | N/A | 33 | F | yes | yes | yes |
| Healthy donor | HD2 | N/A | - | N/A | N/A | N/A | N/A | yes | yes | yes |
| Healthy donor | HD3 | N/A | - | N/A | N/A | N/A | N/A | yes | yes | yes |
| Healthy donor | HD4 | N/A | - | N/A | N/A | N/A | N/A | yes | yes | yes |
| Healthy donor | HD5 | N/A | - | N/A | N/A | N/A | N/A | yes | yes | yes |
| Healthy donor | HD6 | N/A | - | N/A | N/A | 71 | M | yes | yes | yes |
| Healthy donor | HD7 | N/A | - | N/A | N/A | 62 | F | yes | yes | yes |
| Healthy donor | HD8 | N/A | - | N/A | N/A | 65 | M | yes | yes | yes |
| Healthy donor | HD9 | N/A | - | N/A | N/A | 36 | M | yes | yes | no |
| Healthy donor | HD10 | N/A | - | N/A | N/A | 38 | F | yes | yes | no |
| Healthy donor | HD11 | N/A | - | N/A | N/A | 51 | F | yes | yes | no |
| Healthy donor | HD12 | N/A | - | N/A | N/A | 61 | M | yes | yes | no |

* Serological assays were performed on these follow-up samples; however, only the first sample collected from each patient were included in the group-wise analyses and figures.

** Age and sex information for the APS-1 cohort were not reported to protect patient confidentiality. NR, not reported.
