# Supplementary material for: Cellular origin and characterization of autoantibodies against type I IFNs in COVID-19 and APS-1
Source: J Exp Med. 2026 Nov 3;223(12):e20260268. doi: 10.1084/jem.20260268 (PMC13729907; doi:10.1084/jem.20260268)
Supplement: Supplementary file 3 [file jem_20260268_tables5.docx]

**Table S5. Key reagents and resources**

| REAGENT or RESOURCE | Concentration used | SOURCE | IDENTIFIER |
| --- | --- | --- | --- |
| Antibodies | | | |
| FITC anti-human CD3 | 1:1000 dilution | BD Biosciences | Cat# 561800, RRID:AB_10895381 |
| FITC anti-human CD14 | 1:1000 dilution | BD Biosciences | Cat# 569260, RRID:AB_3684914 |
| FITC anti-human CD16 | 1:1000 dilution | BD Biosciences | Cat# 568289, RRID:AB_3684152 |
| PE-Cy7 anti-human CD19 | 1:500 dilution | BD Biosciences | Cat# 560911, RRID:AB_10562390 |
| Fc block | 1:40 dilution | BD Biosciences | Cat# 564219 |
| TotalSeq C0251 Hashtag antibody | 1:100 dilution | BioLegend | Cat# 394661 |
| TotalSeq C0252 Hashtag antibody | 1:100 dilution | BioLegend | Cat# 394663 |
| TotalSeq C0253 Hashtag antibody | 1:100 dilution | BioLegend | Cat# 394665 |
| TotalSeq C0254 Hashtag antibody | 1:100 dilution | BioLegend | Cat# 394667 |
| TotalSeq C0255 Hashtag antibody | 1:100 dilution | BioLegend | Cat# 394669 |
| TotalSeq C0256 Hashtag antibody | 1:100 dilution | BioLegend | Cat# 394671 |
| TotalSeq C0257 Hashtag antibody | 1:100 dilution | BioLegend | Cat# 394673 |
| TotalSeq C0258 Hashtag antibody | 1:100 dilution | BioLegend | Cat# 394675 |
| HRP-conjugated goat anti-human IgG (H+L) | 1:15000 dilution | Thermo Fisher Scientific | Cat# A18805, AB_2535582 |
| Bacterial and virus strains | | | |
| DH5α Competent Cells | | Invitrogen | Cat# 18258012 |
| Biological samples | | | |
| PBMCs and serum from aIFN^+^ severe COVID-19 patients | | Strunz et al., 2024 | Tables S1 and S2 |
| PBMCs and plasma from APS-1 patients | | This paper | Tables S1 and S2 |
| Healthy donor buffy coats | | This paper | Tables S1 and S2 |
| PBMCs and plasma from aIFN^-^ severe/ mild COVID-19 patients | | Marcotte et al., 2022 | Tables S1 and S2 |
| PBMCs and plasma from healthy donors (negative controls) | | This paper | Tables S1 and S2 |
| Chemicals, peptides, and recombinant proteins | | | |
| Recombinant human IFN-α2 | | GenScript | Cat# Z03003 |
| Avi-tagged recombinant human IFN-α2 | | Sino Biological | Custom |
| Streptavidin-R-PE | | BioLegend | Cat# 405204 |
| Streptavidin-APC | | BioLegend | Cat# 405243 |
| NeutrAvidin | | Thermo Fisher Scientific | Cat# 10443985 |
| Fixable Aqua Dead Cell Stain Kit (1:1000 dilution) | | Invitrogen | Cat# L34957 |
| 7-AAD (1:20 dilution) | | BD Biosciences | Cat# 559925 |
| Polyethylenimine (PEI) | | Polysciences | Cat# 24765-1 |
| Tetramethylbenzidine (TMB) substrate | | Sigma-Aldrich | Cat# T0440 |
| QUANTI-Blue substrate | | InvivoGen | Cat# req-qbs |
| DMEM +GlutaMax | | Gibco | Cat# 61965-026 |
| RPMI medium 1640 | | Gibco | Cat# 61870-010 |
| FBS | | Gibco | Cat# 11570506 |
| Penicillin–streptomycin | | Gibco | Cat# 15140122 |
| Critical commercial assays | | | |
| Biotin (Type A) Fast Conjugation Kit | | Abcam | Cat# ab201795 |
| Chromium Next GEM Single Cell 5′Reagent Kit v2 | | 10x Genomics | Cat# PN-1000263 |
| Chromium Single Cell Human BCR Amplification Kit | | 10x Genomics | Cat# PN-1000253 |
| Protein G Sepharose 4 Fast Flow | | GE Healthcare | Cat# 45-000-117 |
| Zeba Spin Desalting Columns | | Thermo Fisher Scientific | Cat# 10280584 |
| Amicon® Ultra Centrifugal Filter, 30 kDa MWCO | | Millipore | Cat# UFC803024 |
| HEK-Blue™ IFN-α/β Reporter Assay | | InvivoGen | Cat# hkb-ifnabv2 |
| Experimental models: Cell lines | | | |
| HEK293FT | | Thermo Fisher Scientific | Cat# R70007 |
| ExpiCHO-S | | Thermo Fisher Scientific | Cat# A29127 |
| IFN-α/β Reporter HEK293 cells | | InvivoGen | Cat# hkb-ifnabv2-a |
| Software and algorithms | | | |
| Cell Ranger v.7.0.0 | | 10x Genomics | <https://10xgenomics.com/> |
| Python v.3.9.19 | | Python Software Foundation | https://www.python.org |
| Scanpy v.1.9.5 | | Virshup et al., 2023 | https://github.com/scverse/scanpy |
| Seurat v.5.1.0 | | [Hao](https://www.nature.com/articles/s41587-023-01767-y#auth-Yuhan-Hao-Aff1-Aff2), et al., 2024 | <https://satijalab.org/seurat> |
| R v.4.3.1 | | R Core Team | https://www.r-project.org |
| Harmony v.1.2.0 | | [Korsunsky](https://pubmed.ncbi.nlm.nih.gov/?term=Korsunsky+I&cauthor_id=31740819) et al., 2019 | <https://github.com/immunogenomics/harmony> |
| ROGUE v.1.0 | | Liu et al., 2020 | <https://github.com/PaulingLiu/ROGUE> |
| Monocle3 v.1.0.0 | | [Cao](https://pubmed.ncbi.nlm.nih.gov/?term=Cao+J&cauthor_id=30787437) et al., 2019 | https://github.com/cole-trapnell-lab/monocle3 |
| Change-O v.1.3.0 | | [Gupta](https://pubmed.ncbi.nlm.nih.gov/?term=Gupta+NT&cauthor_id=26069265) et al., 2015 | <https://changeo.readthedocs.io/> |
| SHazaM v.1.2.0 | | [Gupta](https://pubmed.ncbi.nlm.nih.gov/?term=Gupta+NT&cauthor_id=26069265) et al., 2015 | <https://shazam.readthedocs.io/> |
| R v.4.4.2 | | R Core Team | https://www.r-project.org/ |
| Prism v. 10.2 | | GraphPad | https://www.graphpad.com/ |
