# Supplementary material for: Cellular origin and characterization of autoantibodies against type I IFNs in COVID-19 and APS-1
Source: J Exp Med. 2026 Nov 3;223(12):e20260268. doi: 10.1084/jem.20260268 (PMC13729907; doi:10.1084/jem.20260268)
Supplement: Supplementary file 4 [file jem_20260268_tables6.docx]

List of Karolinska KI/K COVID-19 Study Group consortium authors

Anders Sönnerborg^1^, Dorota Religa^2^, Susanna Brighenti^3^, Benedict J Chambers^3^, Martin Cornillet^3^, Angelica Cuapio^3^, Malin Flodström-Tullberg^3^, Nicole Marquardt^3^, Jagadeeswara Rao Muvva^3^, Anna Norrby-Teglund^3^, Johan K Sandberg^3^, David Wullimann^3^, Isabel Diaz Lozano^4^

^1^Department of Infectious Diseases, Karolinska University Hospital, Stockholm, Sweden

^2^Theme Aging, Karolinska University Hospital, Stockholm, Sweden

^3^Center for Infectious Medicine, Department of Medicine Huddinge, Karolinska Institutet, Karolinska University Hospital, Stockholm, Sweden

^4^UCAM-SENS, Universidad Católica San Antonio de Murcia, UCAM HiTech, Murcia, Spain
